# Highly localized, efficient, and rapid photothermal therapy using gold nanobipyramids for liver cancer cells triggered by femtosecond laser

**DOI:** 10.1038/s41598-023-30526-x

**Published:** 2023-02-27

**Authors:** Xiao Liu, Wei Zhou, Tianjun Wang, Sen Miao, Sheng Lan, Zhongchao Wei, Zhao Meng, Qiaofeng Dai, Haihua Fan

**Affiliations:** 1 Guangdong Provincial Key Laboratory of Nanophotonic Functional Materials and Devices, School of Information and Optoelectronic Science and Engineering, South China Normal University, Guangzhou, 510006 China; 2 School of Biological Science and Medical Engineering, Southeast University, Nanjing, 210096 China; 3 Guangdong Women and Children Hospital, Guangzhou, 51000 China

**Keywords:** Nanoscience and technology, Nanobiotechnology, Nanoparticles, Biomaterials, Nanoscale materials, Plasma physics, Biomaterials, Nanobiotechnology

## Abstract

In this study, the photothermal effect and up-conversion florescence imaging effect of gold nanobipyramids in liver cancer cells are investigated theoretically and experimentally to explore the photothermal ablation tumor therapy with higher photothermal conversion efficiency, shorter laser action time, smaller action range and lower laser power. The small-size gold nanobipyramids with good biocompatibility and infrared absorption peak located in the first biological window are synthesized. Femtosecond laser is focused on the nanobipyramids clusters in cells and the cells die after being irradiated for 20 s at a power as low as 3 mW. In contrast, the control cells die after irradiation with 30 mW laser for 3 min. The theoretical simulation results show that: under femtosecond laser irradiation, the local thermal effect of gold nanoclusters is produced in the range of hundreds of square nanometers and the temperature rises by 516 °C in 106 picoseconds. This therapy reduces the treatment time to seconds level, and the treatment range to square micrometer level, the power to milliwatt level. In this treatment, cells die by apoptosis rather than necrosis, which reduces inflammation. This result opens up a new way to develop photothermal ablation therapy with less side effects and more minimally invasive.

## Introduction

Cancer has been one of the deadliest diseases for humans in recent decades. Human beings continue to make new attempts in diagnosis and treatment technology to fight cancer. In recent years, gold nanomaterials have become one of the main materials for combined treatment of cancer due to their characteristics of unique surface plasmon resonance^1,2^, low toxicity^3,4^, good water solubility^5,6^ and easy surface functionality^7,8^. Photothermal effect caused by plasma resonance is a hot topic in the application of gold nanoparticles in cancer imaging and treatment^9–11^. Previous studies have included the use of photothermal effects to promote drug release from nanoparticles^12–14^ or the combination of photothermal effects with chemotherapy^15–17^, or use the photothermal effect to create a "fever" like environment^17–21^, so as to increase the temperature of the whole tissue by about tens of degrees, so as to achieve the effect of slowly reducing the tumor volume. For these purposes, the previous treatment using photothermal effect is characterized by the irradiation time of a few minutes to more than ten minutes, the illumination power is basically about hundreds of milliwatts, and the illumination range generally covers a large number of cells, on the level of square centimeter.

For some photothermal tumor treatment methods, such as laser tumor ablation technology, the goal of this technology is to rapidly increase the temperature of the tumor target^22,23^. In addition, it is always expected to achieve the goal of local in situ inactivation of the tumor to avoid damage to normal tissues such as blood vessels near the tumor. Therefore, the development of photothermal therapy with less trauma, less postoperative inflammation and less residual scar has become a research hotspot in recent years. To achieve the above purposes, photothermal therapy is required to achieve significant results in a short time and a small range.

Compared with previous photothermal therapy, gold nanobipyramid (GNB) have great potential in improving the light energy utilization efficiency of cancer ablation. First: the specific heat capacity of gold is very small, and the heating rate is very fast. Secondly, Compared with gold spheres and gold nanorods (GNRs), gold nanobipyramids have stronger surface plasmon resonance effect^24^. Compared with gold nanostars, GNB has better biocompatibility. Due to the anisotropy of GNBs, its longitudinal absorption peak can be adjusted in the near-infrared region of the first biological window to reduce the absorption and scattering of excitation light by biological tissues^25^. Most GNBS previously reported for biological applications are usually larger than 100 nm in size if their absorption peak is located in the first biological window^26,27^. However, the smaller the size of GNB, the easier it is for the particles to be taken up by cells^28^. In conclusion, the combination of small size GNB and laser is expected to achieve minimally invasive treatment with fewer traumas, shorter action time and fewer side effects compared with traditional photothermal therapy, and improve the light energy utilization efficiency of cancer ablation. Therefore, it is necessary to explore the mechanism and experimental phenomenon of the photothermal effect of GNB under ultrafast laser irradiation. However, the related reports are lacking.

In this paper, the photothermal effect of GNBs was studied theoretically and experimentally, and a new photothermal therapy with low laser power, short treatment time and small treatment range was developed. In this work, two small size GNBs were synthesized. They are easily taken up by cells and the surface plasmon resonance absorption peak remains in the first biological window. Under the excitation of infrared laser, upconversion fluorescence imaging of cells co-cultured with GNBs can be observed. And the plasmon resonance effect of gold nanoparticles clusters in cells can rapidly heat organelles and induce cell death. Human hepatoma (HepG2) cells co-cultured with GNBS died after being irradiated with 3 mW femtosecond (fs) laser or 8.4 mW continuous laser for 20 s. The photothermal treatment efficiency of GNB was proved higher than that of GNR. The laser power used in GNB group was much lower than that used to induce cell death in the control group. The Apoptosis time of the cancer cells can be controlled by adjusting light intensity. In this low-power laser photothermal treatment, the cells were induced apoptosis rather than necrosis, which can reduce the inflammatory response caused by cell necrosis. At the same time, the simulation results show that the combination of GNBs and fs laser can achieve rapid (nanosecond level) heating or cooling in a small range (hundreds to thousands and of nm^2^ level). To sum up, this new photothermal therapy combined with GNB and fs laser shortens the treatment time, compresses the treatment range, reduces the damage and side effects caused by photothermal effect, and improves the efficiency of photothermal therapy. It opens up a new way for the application of nanoparticles in tumor therapy.

## Results

### Characteristic of GNRs and GNBs

**Figure 1:**
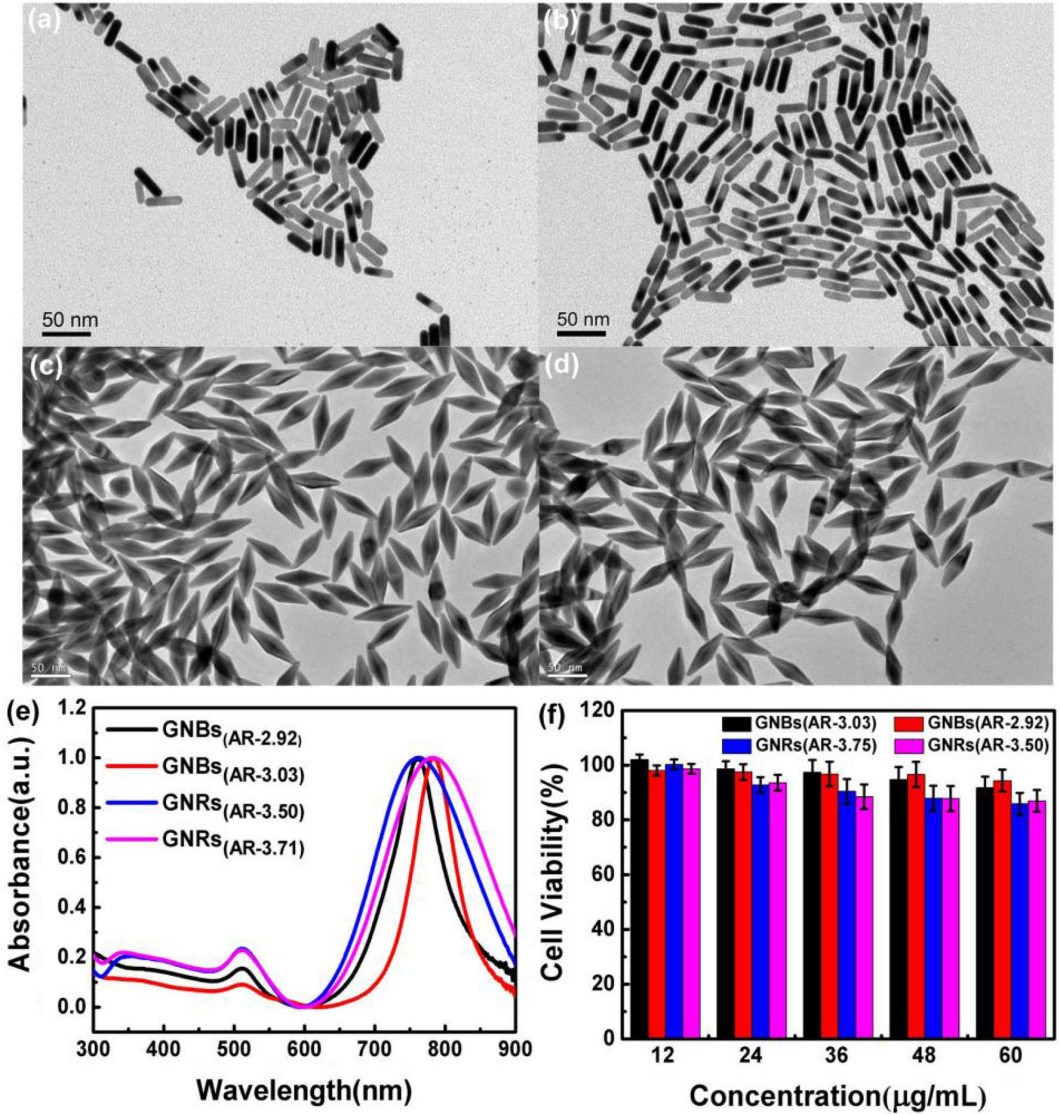
The TEM images of (**a**) GNRs (LSPR 760 nm, AR 3.50); (**b**) GNRs (LSPR 780 nm, AR 3.71); (**c**) GNBs (LSPR 760 nm, AR 2.92 ); and (**d**) GNBs (LSPR 780 nm, AR 3.03). (**e**) UV–Vis absorption spectra of GNRs and GNBs; (**f**) MTT method was used to determine the cytotoxicity of different concentrations of GNRs and GNBs on HepG2 cells. Error bars represent the standard errors of the four experiments.

Since the 700–900 nm band is the first biological window, the absorption of water in this band is negligible^29,30^, and infrared excitation wavelengths can penetrate biological tissue more deeply, gold nanoparticles with absorption peaks at this band were selected as the research object. Based on the aforementioned discussion results, two kinds of GNBs were synthesized by optimizing the synthesis conditions. Fig. S1 shows that the size and absorption peak position of GNBs can be adjusted by changing the experimental conditions. The absorption peaks of GNBs in this work is located in 760 and 780 nm. The size of GNBs is maintained small enough to conducive to cell uptake. For comparison, two GNRs with the same absorption peak as GNBs were also synthesized. Figure 1a–d shows the TEM images of GNBs and GNRs with the longitudinal surface plasmon resonance (LSPR) of 760 and 780 nm, respectively. The shape and size of each GNB particle were highly consistent, with a pointed structure at both ends. The shape was similar to that of cone particles, and the particle size distribution was uniform, indicating that GNB particles with complete shape and structure have been successfully prepared. In Fig. 1c,d, the longitudinal length of GNBs was 65.89 ± 4.02 nm (LSPR ~ 760 nm) and 67.23 ± 3.57 nm (LSPR ~ 780 nm), respectively, and the transverse width was 22.57 ± 1.19 nm (LSPR ~ 760 nm) and 22.25 ± 1.18 nm (LSPR ~ 780 nm) respectively. Fig. S2c and S2d shows the particle size distribution of these two GNBs of different sizes. The length–diameter ratios (the ratio of longitudinal length to transverse width) of these two GNBs were 2.92 ± 0.14 and 3.03 ± 0.17, respectively. The structure and shape of GNRs were complete, and the particle size distribution was uniform. The longitudinal length of GNRs were 33.15 ± 4.56 nm and 35.49 ± 3.89 nm, and the lateral widths were 9.47 ± 1.13 nm and 9.56 ± 0.91 nm, respectively. From the particle size distribution diagram (Fig. S2a and S2b), the aspect ratio (AR) (the ratio of longitudinal length to transverse width) of the two GNRs was 3.50 ± 0.56 nm and 3.71 ± 0.55 nm, respectively.

The absorption spectrum of GNBs and GNRs were measured with an ultraviolet–visible spectrophotometer and showed in Fig. 1e. The absorption spectrum shows that a transverse plasmon resonance absorption peak was observed at 510 nm for both GNBs and GNRs. The longitudinal absorption peaks of GNBs (AR ~ 2.92) and GNRs (AR ~ 3.50) were located at 760 nm. The longitudinal absorption peaks of GNBs (AR ~ 3.03) and GNRs (AR ~ 3.71) were located at 780 nm. The result also indicated the successful synthesis of GNR and GNB particles. In addition, as shown in Fig. S3, when GNB and GNR solutions are excited by fs pulse laser with the same wavelength as their corresponding longitudinal absorption peak, upconversion fluorescence spectra of gold nanoparticles can be obtained.

Good biocompatibility is the key factor for gold nanomaterials to be used in biological therapy. The HepG2 cells were incubated with different concentrations of GNRs and GNBs for 24 h, and the 3-(4,5-dimethylthiazol-2-yl)-2,5-diphenyltetrazolium bromide assay was used to measure the cell viability. The results are shown in Fig. 1f. When the concentration reached 60 μg/mL, the cell viability of GNBs with an AR of 2.92 and 3.03 was 92% and 94%, respectively. The cell viability of GNRs with an AR of 3.50 and 3.71 was 86% and 87%, respectively. These results indicated that GNRs and GNBs modified by PEG were low toxic to cells.

### Interaction between the gold nanoparticles (GNPs) and HepG2 cells

**Figure 2:**
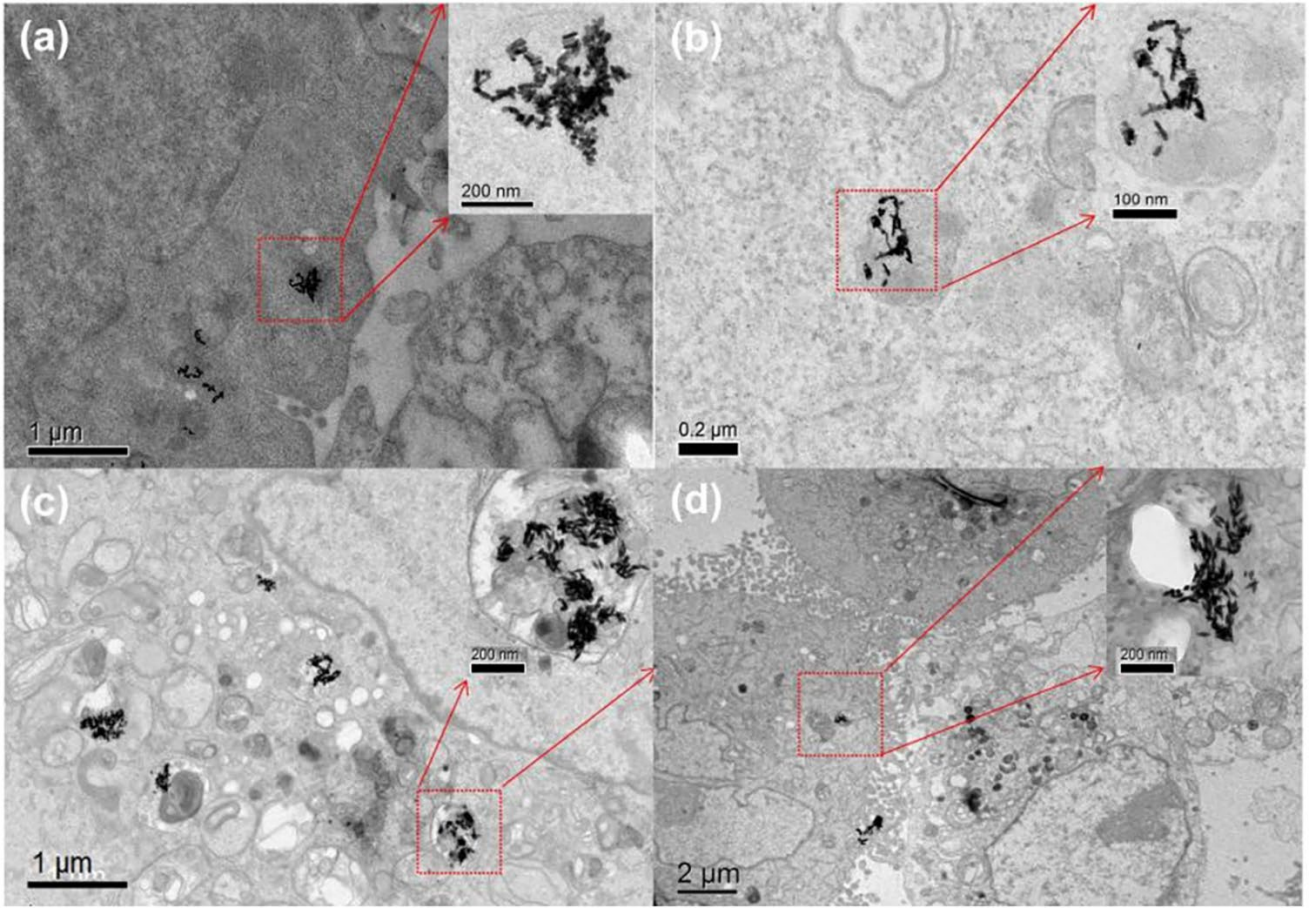
(**a**–**d**) TEM images of HepG2 cells incubated with GNPs for 24 h. (**a**,**b**) TEM images of cells after taking GNRs with an AR of 3.50 and 3.71 respectively; (**c,d**) TEM images of cells after taking GNBs with an AR of 2.92 and 3.03 respectively.

Seyed et al.^31^ showed that particles with a size between 50 and 200 nm were mainly absorbed by clathrin-mediated endocytosis. The content of Au in the cells was measured using ICP-MS and TEM to study the uptake ability of cells to GNBs and GNRs. Table 1 lists the cellular uptake of GNBs and GNRs of different sizes when they are co-cultured with the cells in the concentration of 60 μg/mL. The cellular uptake of GNRs with an AR of 3.50 and 3.71 was 2240/cell and 1880/cell, respectively. The cellular uptake of GNBs with an AR of 2.92 and 3.03 was 1440/cell and 1339/cell, respectively. Table 1 shows that HepG2 cells have a high uptake for both GNBs and GNRs. Moreover, the gold nanoparticles had the same morphology and structure, the small size of gold nanoparticles was easier to be taken up into cells. The TEM images of cell sections were conducted to observe the distribution of GNRs and GNBs in the cells after cultured with the cells. As shown in Fig. 2, after 24 h of incubation of gold nanoparticles with cells, the internalized GNBs and GNRs were distributed in the cytoplasm and around the nucleus, and aggregated in the endocytic vesicles, lysosomes or cytoplasm of the cells. Nanoparticle clusters are formed in cells.

**Table 1:** Uptake of GNRs and GNBs of different sizes by a single HepG2 cell.

Sample	GNRs_(AR-3.50)_	GNRs_(AR-3.71)_	GNBs_(AR-2.92)_	GNBs_(AR-3.03)_
Number of GNPs/Cell	2240	1880	1440	1339

### Upconversion excitation bioimaging

**Figure 3:**
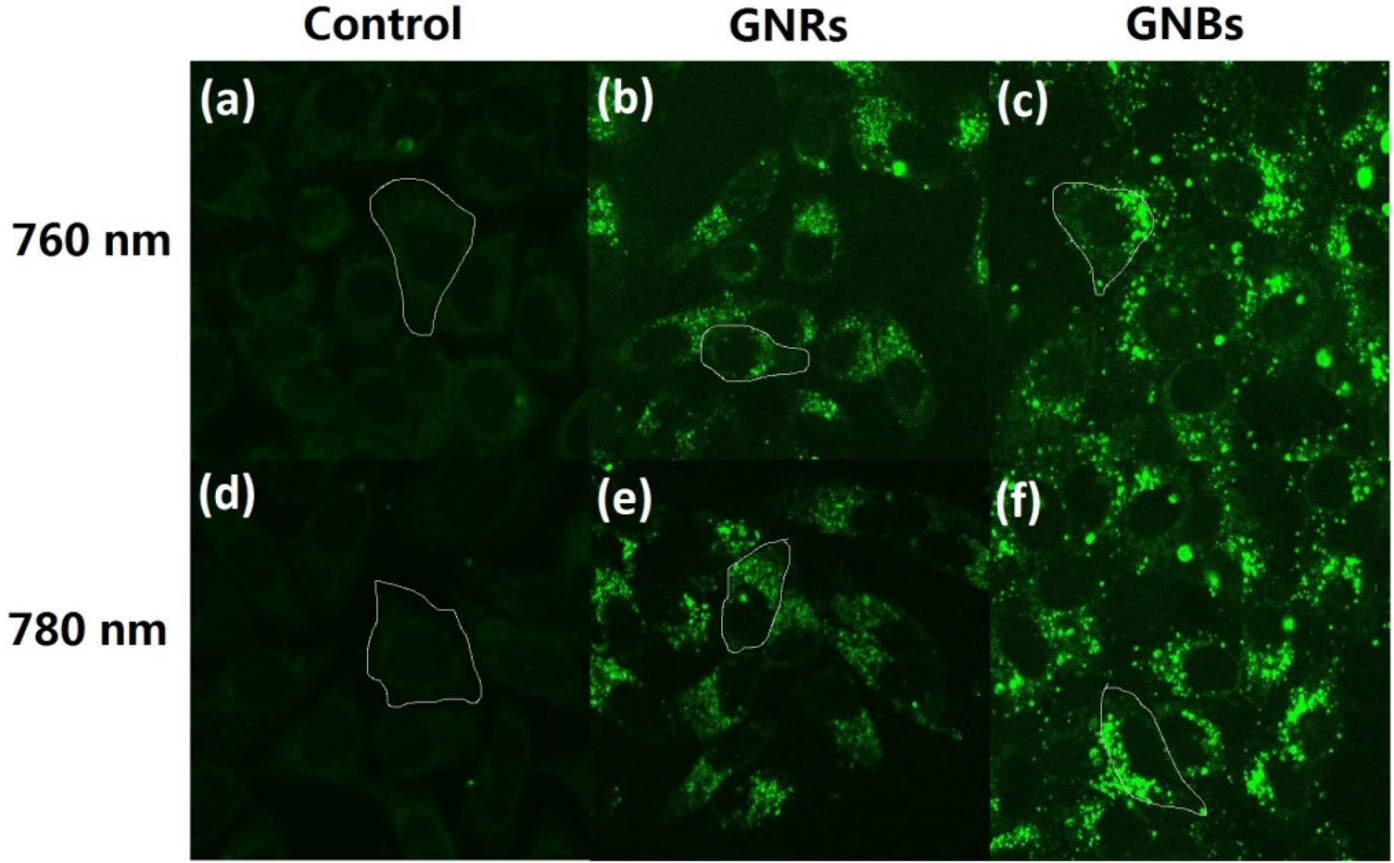
The fluorescence images HepG2 cells under the action of 760 nm fs laser, the selected cell is indicated by white line: (**a**) control group; (**b**) cells incubated with GNRs (AR-3.50) for 24 h; (**c**) cells incubated with GNBs (AR-2.92) for 24 h. The fluorescence images HepG2 cells under the action of 780 nm fs laser: (**d**) control group; (**e**) cells incubated with GNRs (AR-3.71) for 24 h; (**f**) cells incubated with GNBs (AR-3.03) for 24 h.

When the infrared fs laser was used to excite cells co-cultured with gold nanoparticles, upconversion fluorescence images of cells could be obtained due to light scattering and upconversion luminescence properties of gold nanoparticles. By observing the upconversion fluorescence image, the location of the gold nanoparticle clusters could be determined so that the gold nanoparticle clusters could be further excited by an infrared laser for photothermal treatment. First, gold nanoparticles with a concentration of 60 μg/mL were incubated with HepG2 cells for 24 h. Then, the cells were scanned at low power with a fs pulsed laser with the same wavelength as the longitudinal absorption peak of GNBs and GNRs. Therefore, the upconversion fluorescence images could be obtained. Figure 3 shows upconversion fluorescence images of the control group (the cells incubated without gold nanoparticles) and the experiment group (the cells incubated with gold nanoparticles). Combining the TEM image (Fig. 2) and the fluorescence spectrum of gold nanoparticles (Fig. S3) under fs laser excitation, the position of gold nanoclusters can be determined by observing the upconversion fluorescence enhancement point.

The fluorescence intensity values of the selected cells in Fig. 3 are listed in Table 2. The fluorescence intensity of the experimental group cells co-incubated with GNBs and GNRs was significantly higher than that of the control group. The average fluorescence intensity of the experimental group cells was about 3 (GNBs group) and 2 (GNRs group) times that of the control group cells, respectively. The comparison of the fluorescence intensity of the two groups of experimental cells showed that GNBs and GNRs could effectively enhance the fluorescence intensity of HepG2 cells and GNBs had better cell fluorescence imaging effects than GNRs. These results have guiding significance for future biological cell imaging and fluorescent labeling.

**Table 2:** The fluorescence brightness of the selected cell.

Sample	(a) control	(b) GNRs	(c) GNBs	(d) control	(e) GNRs	(f) GNBs
Area (cell)	1	1	1	1	1	1
Intensity (min)	3	4	3	3	2	2
Intensity (max)	22	104	127	21	108	128
Intensity (mean)	8.4	17.7	28.9	6.5	17.1	30

## Photothermal therapy of GNRs and GNBs

### Photothermal treatment effect of GNRs and GNBs under pulsed laser irradiation

In this work, the position of the gold nanorod cluster in the cell could be obtained by fast scanning the cell with a low power 800-nm laser and observing the up-conversion fluorescence emission image of the cell. After determining the location of the GNP cluster, a high-power femtosecond laser could be used to simply excite the GNP clusters. Due to the high peak power and short pulse time of fs pulse, the gold nano particle clusters in the cell can be irradiated in a very short time, so that the clusters can obtain high local energy and rapid heating. The schematic diagram of the experimental device is shown in Fig. S4. The laser is focused on the cell organelles containing the nanoclusters through the 60× microscopic objective, and the spot diameter is about 1 μm. In this way, an accurate and rapid injury to a single cell can be easily achieved. Fig. S5 shows the process of bubbles generated by rapid heating of gold nanoclusters under fs laser irradiation. In this study, gold nanoparticles of different sizes were used as light-to-heat conversion agents to study the photothermal treatment for HepG2 cells under pulsed laser irradiation. All cells were irradiated directed at intracellular nanoclusters for 20 s with fs laser pulse of varying power. The heat generated by the clusters was different, and the effect on the cells was different due to the different powers of laser irradiation. The timing of cell death could be controlled by varying the laser power. The cells were stained with trypan blue dye to determine whether they were dead after the laser treatment^32^.

**Figure 4:**
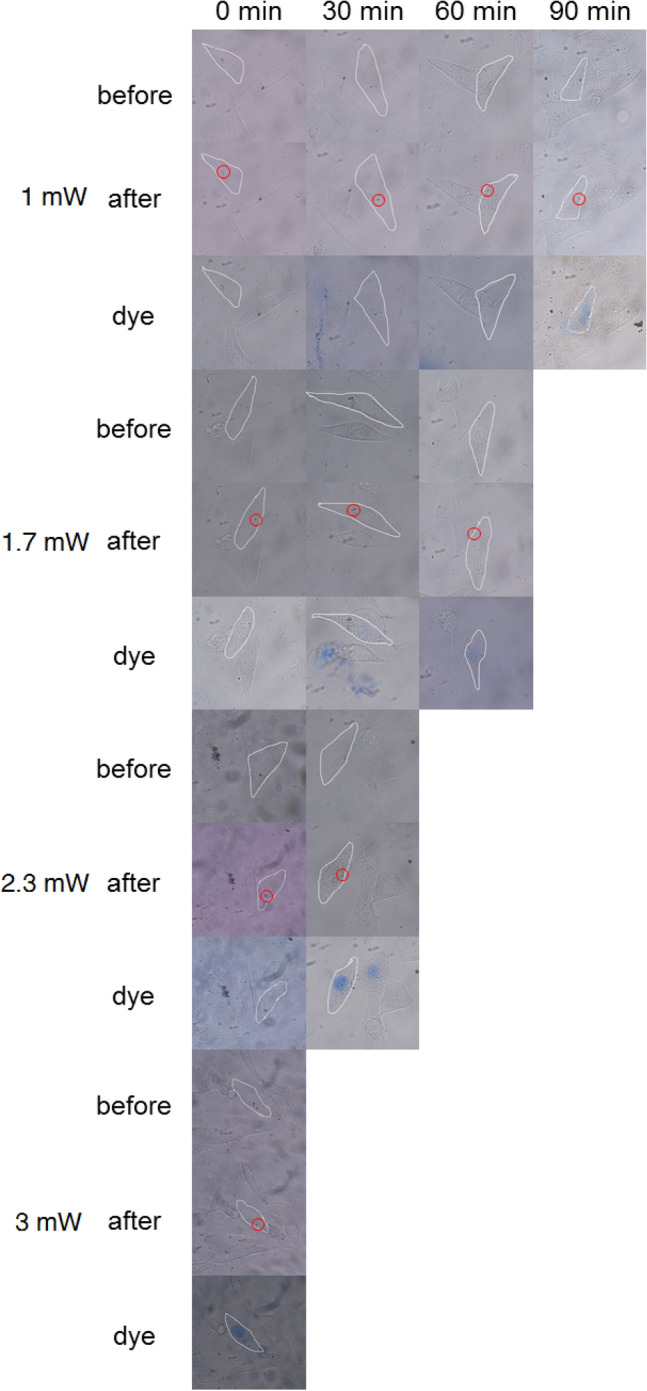
Photothermal treatment of GNBs with an AR of 2.92 under 760 nm fs pulsed laser, the laser focuses on the nano clusters in the cell. The red circle in the figure marks the position of the laser. The selected cell is indicated by white line.

Figure 4 and Fig. S6 illustrate the photos of HepG2 cells cultured with GNBs (LSPR, 760 nm) and GNRs (LSPR, 760 nm) after fs pulsed laser irradiation with the different power. Fig. S7 and S8 show the images of cells co-cultured with GNBs (LSPR, 780 nm) and GNRs (LSPR, 780 nm) after pulsed laser irradiation with different powers. The cell death times for GNBs and GNRs of different sizes under different powers are listed in Table 3 to compare the photothermal treatment effects of the gold nanoparticles. Figure 4 and Fig. S6–S8 and Table 3 demonstrate that when GNBs were used as photothermal treatment agents, Lower laser power is required to induce cell death. For example, when the pulsed laser power was as low as 3 mW, the cells of the GNB experimental group died immediately. However, for the GNR experimental group, the cell died 30 min later. When the incident laser power was 1 mW, the cells of the GNB experimental group died 90 min after laser irradiation, while cells of the GNR experimental group died 120 min after laser irradiation. For comparison, the cells cultured without gold nanoparticles were irradiated with a pulse laser. The experimental results are shown in Fig. S9. After irradiation with a 3 mW pulsed laser, the cells had no obvious damage and the cells still alive after 120 min. In addition, when the power of the pulsed laser was increased to 30 mW, the blank cells died after being irradiated by the laser for 3 min. These results indicated that (1) the combination of gold nanoparticles and fs laser can rapidly induce cancer cell death; (2) GNBs had a better photothermal treatment effect than GNR particles.

**Table 3:** Summary of the information about the fs laser power and cell death time.

Sample	Death time				
	1 mW (min)	1.7 mW (min)	2.3 mW (min)	3 mW (min)	4 mW (min)
GNBs-760 nm	90	60	30	0	–
GNRs-760 nm	120	90	60	30	0
GNBs-780 nm	90	60	30	0	–
GNRs-780 nm	120	90	60	30	0

### Photothermal treatment effect of GNRs and GNBs under continuous laser irradiation

**Figure 5:**
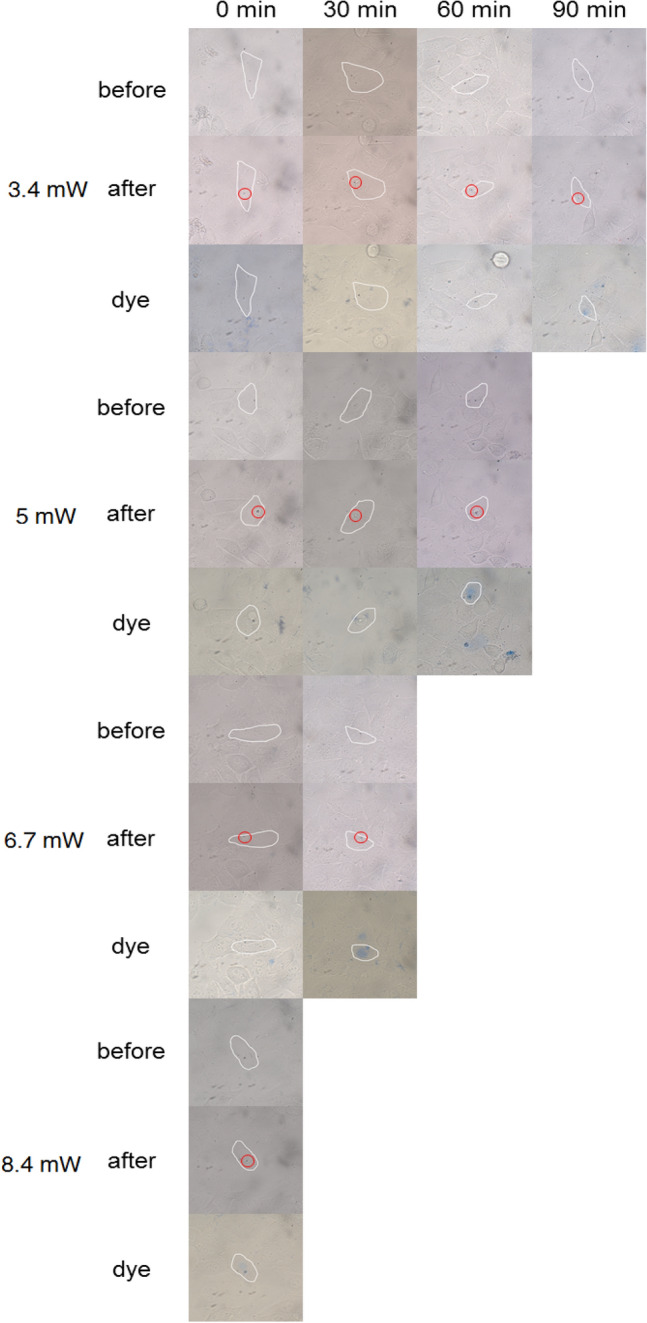
Photothermal treatment of GNBs with an AR of 2.92 under 760 nm continuous laser, the laser focuses on the nano clusters in the cell. The red circle in the figure marks the position of the laser. The selected cell is indicated by white line.

In addition to the photothermal experiment with fs laser, the photothermal effect under continuous light excitation has also been studied. Due to the strong plasma resonance effect of GNBs, continuous laser irradiation on GNBs clusters can also produce high temperature. Under this high temperature condition, cancer cells can also be effectively induced to die. Moreover, compared with a fs laser, the price of a continuous wave laser is much cheaper. Therefore, it is of great significance to study the response of cells co-cultured with gold nanoparticles under continuous light irradiation. Figure 5 and Fig. S10–Fig. S12 respectively show the photothermal treatment effect of HepG2 cells after continuous irradiation of gold nanoclusters in cells with 760 nm and 780 nm laser for 20 s. Tables 4 and 5 briefly list the death time of cells co-cultured with GNBs and GNRs after different power laser treatment. The results show that when the continuous laser power was 8.4 mW, the cancer cells cultured with GNBs died immediately. Even the use of continuous laser irradiation with a power as low as 3.4 mW could cause cell death 90 min later. For the experimental group of GNR, the laser power of inducing cell death was higher than that of the GNB group. The experimental results of HepG2 cells without gold nanoparticles irradiated with 25 mW continuous laser for 5 min are shown in Fig. S13. The cells had no obvious damage and did not die after 90 min.

**Table 4:** Summary of the information about the continuous laser power and cell death time (GNB).

Sample	Death time			
	3.4 mW (min)	5 mW (min)	6.7 mW (min)	8.4 mW (min)
GNBs-760 nm	90	60	30	0
GNBs-780 nm	90	60	30	0

**Table 5:** Summary of the information about the continuous laser power and cell death time (GNR).

Sample	Death time			
	6.7 mW (min)	8.4 mW (min)	11 mW (min)	14 mW (min)
GNRs-760 nm	90	60	30	0
GNRs-780 nm	90	60	30	0

### The form of cell death

**Figure 6:**
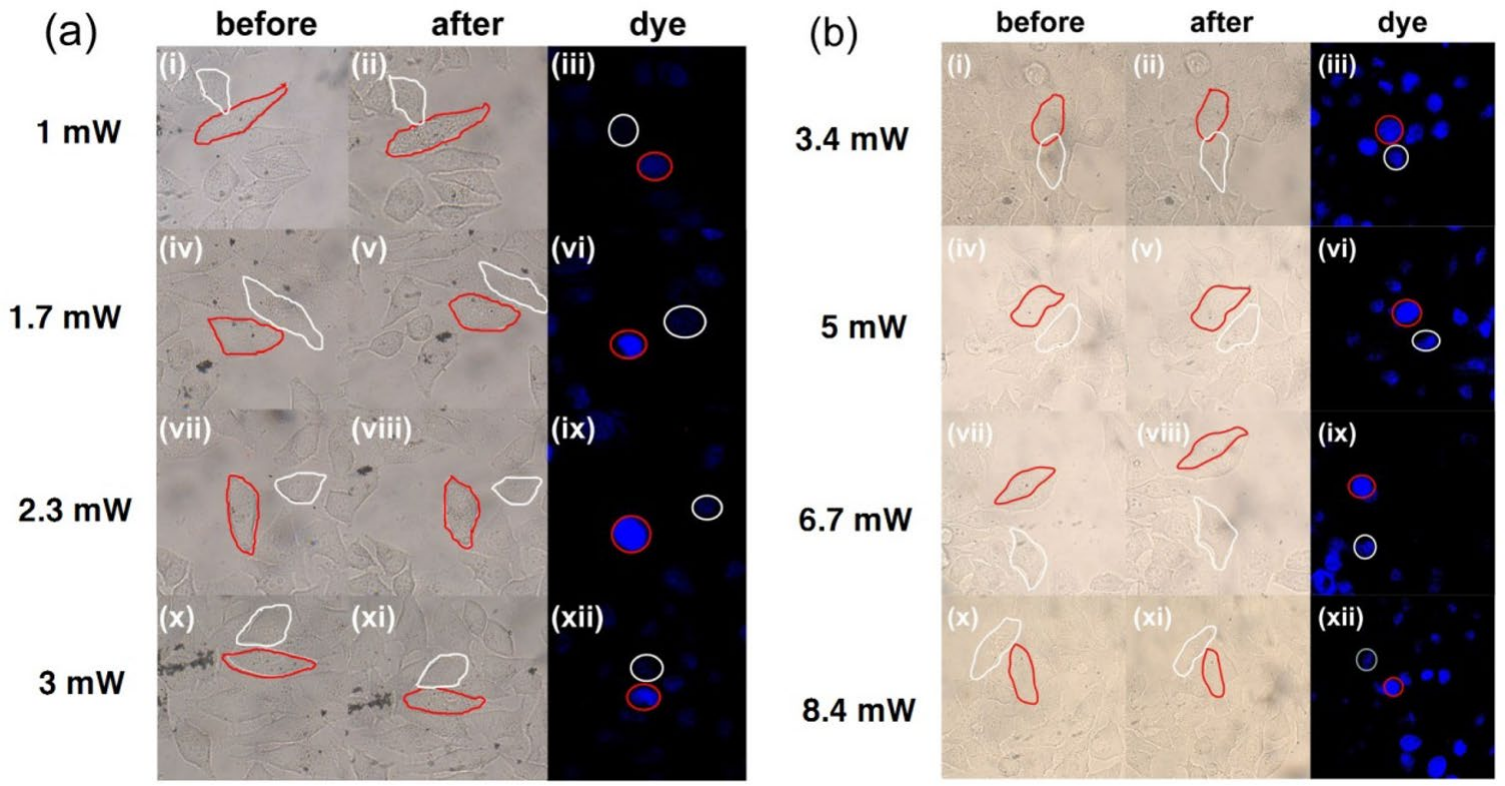
(**a**) shows GNBs with an AR of 2.92 induce cell apoptosis under 760 nm fs pulsed laser irradiation. (**b**) GNBs with an AR of 2.92 induce cell apoptosis under 760 nm continuous laser irradiation. The white circles represent control cells and the red circles represent experimental cells.

In addition to studying the photothermal effect of laser on cells co-cultured with gold nanoparticles, the form of cell death induced by photothermal effect was also studied due to its significance for clinical application. The main form of cell death were apoptosis, necrosis, and programmed death. The dye Hoechst33342 and propidium iodide (PI) were used to determine whether the cell death form was apoptosis or necrosis in this work. After the cells co-cultured with nanoparticles were irradiated with pulse lasers of different powers, they were stained with Hoechst33342 dye solution for 15 min in the dark. Then the staining of the cells was observed under ultraviolet light irradiation to determine the dead form of cells which were irradiated by the laser. The experimental results of fs laser treatment groups are shown in Fig. 6a and Fig. S14–S16. The nuclei of target cells had bright blue fluorescence (apoptosis), while the nuclei of control cells (without laser treatment) had weak blue fluorescence (live). The fluorescence intensity of the control cells and target cells is listed in Table 6 and Table S1–S3, respectively. The blue fluorescence intensity of target cells after fs laser treatment was found to be significantly higher than that of control cells. In addition, the form of cell death induced by continuous lasers was also studied. The cells co-cultured with GNBs were stained after being treated with continuous lasers of different powers. The experimental results are shown in Fig. 6b and S17, and the corresponding fluorescence intensity values are shown in Table 7 and Table S4, respectively. The analysis of the experimental results indicated that compared with the control cells, the nucleus of the target cells showed bright blue fluorescence, and the fluorescence intensity value was significantly higher than that of the control cells. The aforementioned analysis indicated that the cells cultured with gold nanoparticles were induce apoptosis by both the low-power fs pulsed laser and the continuous laser. However, when the PI dye was used, the laser-treated cells co-cultured with gold nanoparticles could not be stained and no bright red was observed, which proved that the way these cells died was not necrosis, but apoptosis. After laser treatment for cells, it was likely that only the organelles near the nanoclusters in cells were damaged due to the small range and short time of photothermal effect, while the cell membrane remained intact. Therefore, as long as the power and time of laser irradiation on the cells are well controlled, apoptosis can be triggered and cell necrosis can be avoided, thereby avoiding inflammation caused by cell necrosis.

**Table 6:** Fluorescence intensity values of control cells and experimental cells (incubate with GNBs with an aspect ratio of 2.92) in Fig. 6a.

Sample	(iii)_Control_	(iii)_GNBs_	(vi)_Control_	(vi)_GNBs_	(ix)_Control_	(ix)_GNBs_	(xii)_Control_	(xii)_GNBs_
Area (cell)	1	1	1	1	1	1	1	1
Intensity (average)	19.359	49.754	43.992	74.759	48.545	81.981	35.813	58.362

**Table 7:** Fluorescence intensity values of control cells and experimental cells (incubate with GNBs with an aspect ratio of 2.92) in Fig. 6b.

Sample	(iii)_Control_	(iii)_GNBs_	(vi)_Control_	(vi)_GNBs_	(ix)_Control_	(ix)_GNBs_	(xii)_Control_	(xii)_GNBs_
Area (cell)	1	1	1	1	1	1	1	1
Intensity (average)	68.775	83.187	58.057	79.338	55.945	81.064	68.256	83.611

## Discussion

### Photothermal effect and field enhancement of GNRs and GNBs

After obtaining a series of experimental results, in order to further analyze the experimental results and explore the mechanism of heat generation of gold nanoparticles under laser irradiation, COMSOL software was used for numerical simulation. Mathematical models were established to study the field enhancement and temperature distribution around gold nanoparticles excited by near-infrared laser.

When the gold nanoparticles were excited by continuous Gaussian light, the photothermal process could be described using the following equations^33,34^:

1 $$\nabla \times\upmu _{{\text{r}}}^{ - 1} \left( {\nabla \times {\text{E}}} \right) - {\text{k}}_{0}^{2} \left( {\upvarepsilon _{{\text{r}}} - \frac{{{\text{j}}\upsigma }}{{\upomega \upvarepsilon _{0} }}} \right){\text{E}} = 0$$

2 $${\text{qr}} = \frac{1}{2}\upvarepsilon _{0}\upomega {\text{lm}}\left( {\upvarepsilon _{{\text{r}}} } \right)\left| {\text{E}} \right|^{2}$$

3 $$\uprho {\text{c}}_{{\text{p}}} \frac{{\partial {\text{T}}}}{{\partial {\text{t}}}} + \nabla \cdot \left( { - {\text{k}}\nabla {\text{T}}} \right) = {\text{qr}}$$

Equation (1) is the Helmholtz equation in COMSOL Multiphysics 5.6. It was solved to obtain the electric field distribution E. In Eq. (1), ε_0_ is the vacuum permittivity; ω is the angular frequency of input light; k_0_ is the wave vector of the input light; σ is electrical conductivity; μ_r_ is the relative permeability, which, in this case, was 1 both for media (assuming water) around the nanoparticle and gold. ε_r_ is the relative permittivity. For media in this work, it is set to 1.77. For gold, ε_r_ is obtained from Johnson and Christy^35^. Equation (2) was used to solve the heat power volume density, qr is heat power volume density, and lm(ε_r_) is the imaginary part of relative permittivity. The imaginary part of the relative permittivity of media (water) is so small that it can be ignored, as reported in two previous studies^24,25^. Therefore, the imaginary part was set to zero in this simulation. Equation (3) was used to get the temperature distribution in space, where k is the thermal conductivity, ρ is the mass density, and c_p_ is the thermal capacity. For water, k = 0.6 W m^−1^ K^−1^, ρ = 1000 kg m^−3^, and $c_{p}$ = 4180 J kg^−1^ K^−1^. For gold, k = 317 W m^−1^ K^−1^, ρ = 19,300 kg m^−3^, and $c_{p}$ = 129 J kg^−1 ^K^−1^^36^.

When excited by a fs Gaussian pulse laser, the temperature should be considered as three parts: the temperature of water Tm, the temperature of electrons Te in gold nanoparticles, and the temperature of lattice T1 in gold nanoparticles. The three temperatures were described by the “two-temperature model”^37–39^. The corresponding equations were as follows:

4 $${\text{S}}\left( {\text{t}} \right) = \frac{{{\text{qr}}}}{{\sqrt {2\uppi } {\text{t}}_{\upsigma } }}{\text{exp}}\left( {\frac{{ - \left( {{\text{t}} - {\text{t}}_{0} } \right)^{2} }}{{2{\text{t}}_{\upsigma }^{2} }}} \right)$$

5 $${\text{C}}_{{\text{e}}} \frac{{\partial {\text{T}}_{{\text{e}}} }}{{\partial {\text{t}}}} = \nabla \cdot \left( {{\text{k}}_{{\text{e}}} \nabla {\text{T}}_{{\text{e}}} } \right) - {\text{g}}\left( {{\text{T}}_{{\text{e}}} - {\text{T}}_{{\text{l}}} } \right) + {\text{S}}\left( {\text{t}} \right)$$

6 $${\text{C}}_{{\text{l}}} \frac{{\partial {\text{T}}_{{\text{l}}} }}{{\partial {\text{t}}}} = \nabla \cdot \left( {{\text{k}}_{{\text{l}}} \nabla {\text{T}}_{{\text{l}}} } \right) + {\text{g}}\left( {{\text{T}}_{{\text{e}}} - {\text{T}}_{{\text{l}}} } \right) - {\text{G}}\left( {{\text{T}}_{{\text{l}}} - {\text{T}}_{{\text{m}}} } \right)\frac{S}{V}$$

7 $$\uprho _{{\text{m}}} {\text{C}}_{{\text{m}}} \frac{{\partial {\text{T}}_{{\text{m}}} }}{{\partial {\text{t}}}} = \nabla \cdot \left( {{\text{k}}_{{\text{m}}} \nabla {\text{T}}_{{\text{m}}} } \right) + {\text{G}}\left( {{\text{T}}_{{\text{l}}} - {\text{T}}_{{\text{m}}} } \right)\frac{S}{V}$$

In Eq. (4), the qr is obtained from Eq. (2), t_σ_ = t_*l*_/(2$\sqrt {{\text{2ln2}}}$), t_*l*_ is the pulse width, and $t_{0}$ is the position of the center of the peak. In this simulation, $t_{l}$ = 120 fs and $t_{0}$ = 500 fs. In Eqs. (6) and (7), S is the surface of gold nanoparticles and V is the volume of gold nanoparticles. g is the electron–lattice coupling coefficient, which describes the energy exchange from electron to lattice. G is the interface thermal conductivity between lattice and the water, which describes the energy transfer from the lattice to the water. The other parameters were as described in a previous study^32^. The frequency of the fs laser was 76 MHz.

The electric field and temperature distribution of intracellular gold nanoclusters irradiated by continuous and fs pulsed lasers were compared and studied using numerical model above. Based on the particle distribution obtained from the cell TEM images in Fig. 2, numerical simulation models were constructed. After the incident light interacted with the gold nanoparticles, field-enhancement was caused by plasmon resonance coupling. Upon surface plasmon formation, nonradiative relaxation occurs through electron–phonon and phonon–phonon coupling, efficiently generating localized heat that can be transferred to the surrounding environment. Therefore, the temperature of the organelles that enclose the nanoparticles rose, which causes the cell death.

**Figure 7:**
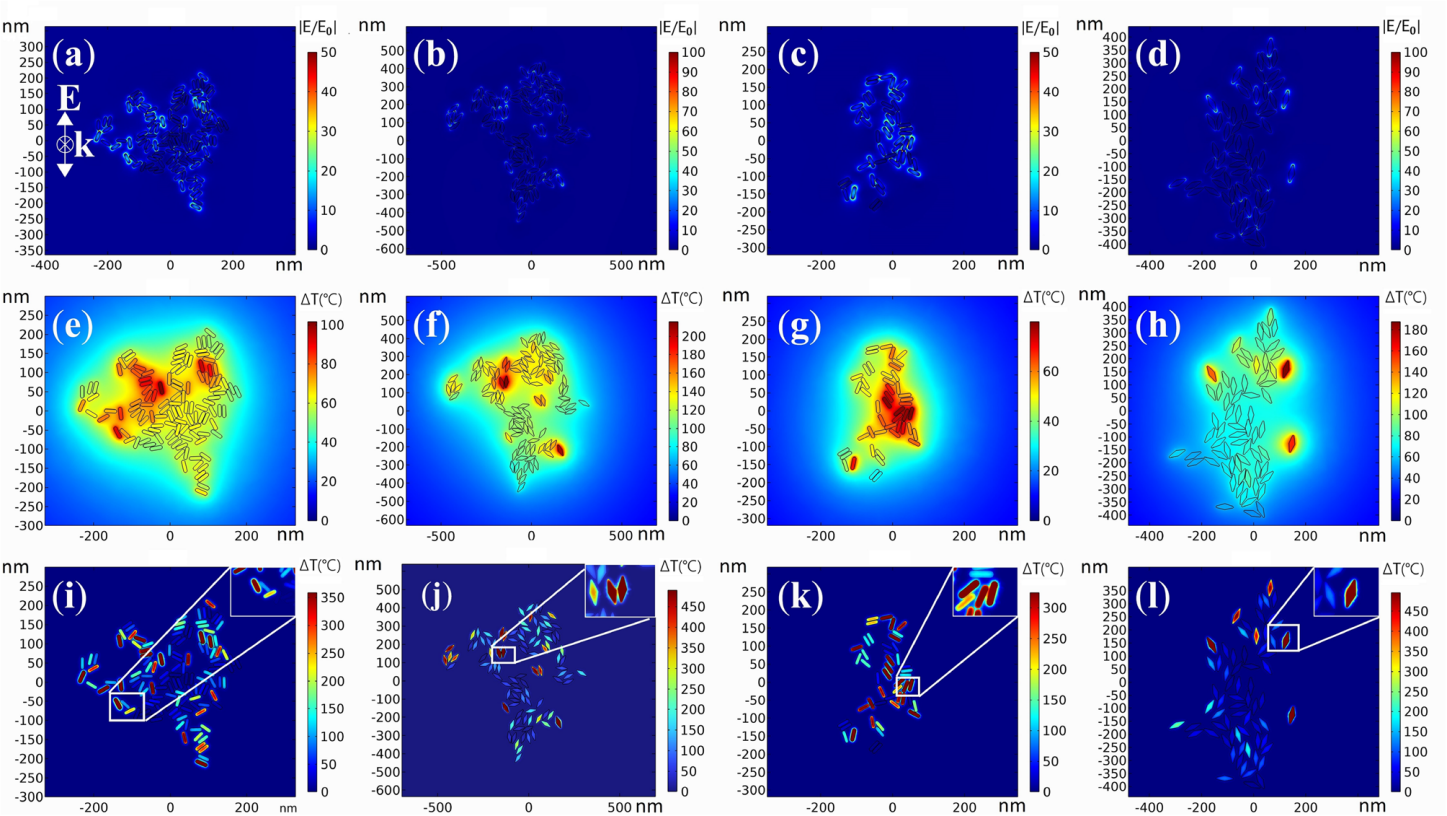
The near-field intensity map of (**a**) GNR (LSPR 760 nm) clusters at 760 nm laser excitation; (**b**) GNB (LSPR 760 nm) clusters at 760 nm laser excitation; (**c**) GNR (LSPR 780 nm) clusters at 780 nm laser excitation; (**d**) GNB (LSPR 780 nm) clusters at 780 nm laser excitation. Rising temperature distribution of: (**e**) GNR (LSPR 760 nm) clusters under 760 nm continuous laser excitation; (**f**) GNB (LSPR 760 nm) clusters under 760 nm continuous laser excitation; (**g**) GNR (LSPR 780 nm) clusters under 780 nm continuous laser excitation; (**h**) GNB (LSPR 780 nm) clusters under 780 nm continuous laser excitation. Rising temperature distribution of (**i**) GNR (LSPR 760 nm) clusters under 760 nm pulse fs laser excitation; (**j**) GNB (LSPR 760 nm) clusters under 760 nm pulse fs laser excitation; (**k**) GNR (LSPR 780 nm) clusters under 780 nm pulse fs laser excitation; (**l**) GNB (LSPR 780 nm) clusters under 780 nm pulse fs laser excitation. The particle distribution in gold nanoclusters in numerical simulation is based on the particle distribution model obtained from the TEM images of cells in Fig. 2.

**Figure 8:**
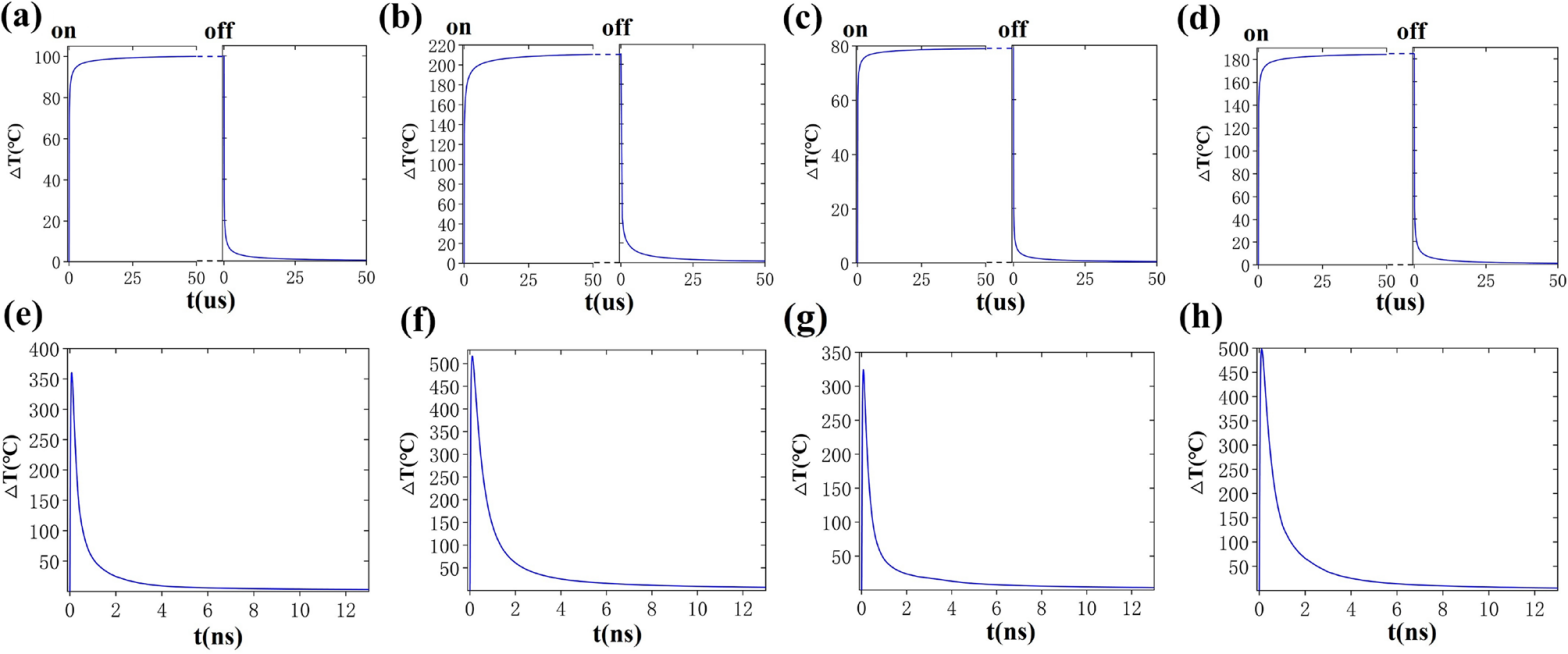
The max transient temperature increasement of gold nanoclusters in water excited by on–off modulated continuous Gaussian light (spot radius = 1000 nm, power = 3.4mW): (**a**) GNR (LSPR 760 nm) clusters at 760 nm laser excitation; (**b**) GNB (LSPR 760 nm) clusters at 760 nm laser excitation; (**c**) GNR (LSPR 780 nm) clusters at 780 nm laser excitation; (**d**) GNB (LSPR 780 nm) clusters at 780 nm laser excitation. The max transient temperature increasement of water around gold nanoparticle clusters excited by fs laser in a pulse period (pulse width t_*l*_ = 120 fs, power = 1mW, pulse frequency = 76 MHz). (**e**) GNR (LSPR 760 nm) clusters at 760 nm laser excitation; (**f**) GNB (LSPR 760 nm) clusters at 760 nm laser excitation; (**g**) GNR (LSPR 780 nm) clusters at 780 nm laser excitation; (**h**) GNB (LSPR 780 nm) clusters at 780 nm laser excitation.

Figure 7a–d show a schematic diagram of field enhancement (|E|/|E0|) of GNB and GNR cluster with the LSPR of 760 nm and 780 nm respectively. The comparison of Fig. 7a,b showed that the field enhancement of GNB (LSPR ~ 760 nm) clusters reached 396, which was much higher than that of GNR with 168 times (Fig. 7a,b). The same phenomenon existed for nanoparticles with LSPR ~ 780 nm (Figs. 7c, 8d). The field enhancement of GNB was larger than GNR. Due to the strong field enhancement effect, GNB has high photothermal conversion efficiency.

The temperature profiles of gold nanoparticle clusters under the laser irradiation were simulated based on equaition (1)-(3) to study the response of gold nanoparticle clusters to the continuous laser. In these simulations, we ignore the influence of the phase transition of the medium in order to simplify the model. Figure 7f shows that the maximum temperature increasement of GNB (LSPR ~ 760 nm) clusters reached 208 °C under the irradiation of 760 nm continuous laser. For GNB clusters, the area warmed by 200 °C is about 2500 nm^2^, and the range warmed by 60 °C is about 1 μm^2^. While the maximum temperature increasement of GNR clusters reached 100 °C under the same conditions. In the case of 780-nm laser irradiation, the temperature rise of GNB clusters was 180 °C, and the temperature rise of GNR clusters was 80 °C. As shown in Fig. 8, the time for heating and cooling of nanoclusters was also studied. Taking GNB (LSPR ~ 760 nm) as an example, under continuous laser irradiation, the particles rose by 196 °C within 4 microseconds (μs) (Fig. 8b), and then the speed of temperature rise slowed down. After 20 μs, the system temperature was basically stable. The temperature of the nanoparticle and the surrounding water rose by 208 °C. Nanocluster can maintain this temperature if irradiated by laser all the time. Moreover, if the continuous laser no longer irradiated the nano clusters, the temperature rapidly dropped to only 12 °C higher than the initial temperature in the first 5 μs, and later the system slowly returned to the original temperature.

In addition to the temperature response of nanoclusters irradiated by a continuous laser, the thermodynamics of gold nanoclusters irradiated by a fs laser was also studied based on Eqs. (4)–(7). In these simulations, the phase transition of the medium is also neglected. Based on the experimental parameters, the laser pulse time was set to 120 femtoseconds and the pulse frequency is 76 MHz. The thermodynamic process of gold nanoclusters irradiated by a fs laser is complex. The most noteworthy thing in the whole system was the temperature of the medium because the temperature of the medium was actually the rising temperature of organelles after the nanoparticles were irradiated by fs laser. Therefore, in Fig. 7i–l, only the maximum temperature distribution of medium after nanoparticles were irradiated by a fs laser is given. Figure 7j show that the medium temperature around the GNB clusters (LSPR, 760 nm) clusters increased by 516 °C. The illustration in Fig. 7j shows the GNB with the highest temperature rise in the whole cluster, which is taken as the research object to study the thermodynamic process. Fig. S18 shows the evolution process of Te, T1, and Tm of this GNB. The calculated results were used to describe the thermal evolution of the system, ignoring the initial nonthermal electron distribution. At the beginning of the pulse, the conduction electrons in the shell first absorbed the laser energy through the excitation of the plasma due to the small heat capacity of the electrons, and the electron temperature increased rapidly to the peak within 120 fs after the pulse peak. Then, the energy accumulated in the electronic system was transferred to the lattice through electron lattice coupling so that the lattice temperature also increased. Finally, through the lattice–lattice interaction, when the gold nanoparticles and the medium around the nanopaticle reached the equilibrium temperature, the heat energy was transferred to the environment. Fig. S18 shows that the electron temperature varied greatly, followed by the lattice and the medium. Gold nanoparticles rose to a higher temperature when a pulsed fs laser was a source comparing with continuous laser (Fig. 7i–l).

Figures 7 and 8 show that the medium temperature around the GNB (LSPR, 760 nm) clusters increased by 516 °C within 0.1 ns under the irradiation of fs 760 nm. This heating rate was much higher than that under continuous laser (198 °C within 5 μs). As showed in Fig. S18, the temperature increasement of the medium around the GNB (LSPR, 760 nm) drop from the peak of 516 °C to 19.6 °C in the 5 ns (Fig. S18). The cooling rate of the medium is also higher than that of continuous light. The temperature of medium around the GNB (LSPR ~ 760 nm) remained 6.8 °C higher than the original temperature before the next pulse arrived for the femtosecond pulse interval is 13 ns. This phenomenon can be attributed to the high localization of heat generated in GNB clusters under fs pulse laser. The heated area of the medium is very small. After the laser is over, the heated part of the medium can quickly reach thermal balance with the surrounding environment. As can be seen from Fig. 7, under fs laser irradiation, the area heated above 50 °C near GNB clusters reached hundreds to thousands of nm^2^. Under continuous laser irradiation, the range of temperature raised above 50 °C near GNB clusters reaches the μm^2^ level, which is about 100 times higher than that under fs laser. This kind of local efficient heating is more conducive to the application of photothermal effect in photothermal therapy. The influence of nanoparticle density on photothermal temperature are also be simulated (Fig. S19). When the number of nanoparticles is within 100, and the research scope is within 1 μm^2^, the maximum temperature increase of water is proportional to the particle density. Whether for continuous laser or pulsed laser.

## Conclusion

In this study, a rapid, efficient and low-cost photothermal therapy with a small action range was developed, and a model was constructed to explore its mechanism. Two kinds of GNBs with absorption peaks located in the first biological window were synthesized in this study. For comparison, two GNRs with the same absorption peak as GNBs were also synthesized. The spectral properties and morphology of four kinds of gold nanoparticles were measured. The cell viability and uptake of gold nanoparticles for HepG2 cells were measured. The results showed that the gold nanoparticles had low cytotoxicity and good biocompatibility. On the basis of this conclusion, the two-photon fluorescence imaging of GNBs and GNRs was compared, and the fluorescence intensity of GNBs in cells was found to be higher than that of GNRs. The photothermal treatment effects of GNRs and GNBs on HepG2 cells were studied systematically in theory and experiment. The theoretical analysis showed that these four gold nanoparticles had different degrees of field enhancement effect and photothermal effect under laser irradiation. The photothermal conversion effect of GNB was stronger than that of GNR. The photothermal effect of nanoparticles excited by a fs laser was stronger than that excited by a continuous laser. The results of photothermal experiments on cells confirmed the aforementioned conclusions. Different power pulses and continuous laser irradiation were used to irradiate nanoclusters in cells, and then the state of cells was observed. The HepG2 cells co-cultured with GNBs were immediately induced to apoptosis after 20 s of pulsed laser irradiation with a power as low as 3 mW or 20 s of continuous laser irradiation with a power as low as 8.4 mW. In this low-power laser photothermal treatment, GNBs and GNRs induced apoptosis rather than necrosis, which greatly avoided the damage to normal tissues caused by the photothermal effect. This study has important guiding significance for developing new cancer ablation technology and the application of gold nanoparticles in biomedicine.

## Methods

### Synthesis of PEG-coated GNRs

GNRs were prepared by referring to the seedless synthesis method reported by El-Sayed et al.^40^, and then polyethylene glycol (PEG) was used to functionalize the prepared GNRs. See Supplementary Material for a description of specific experimental procedures.

### Synthesis of PEG-coated GNBs

GNB particles were prepared by referring to the seed growth method proposed by Li et al.^41^, and then polyethylene glycol (PEG was used to functionalize the prepared GNRs. See Supplementary Material for a description of specific experimental procedures.

### Characterization

A transmission electron microscope (TEM was used to observe the morphology of GNRs and GNBs. The image was taken using a JEM-2100HR (JEOL, Japan) at an accelerating high voltage of 200 kV. An ultraviolet–visible spectrophotometer (UV-2700, Shimadzu, Japan) was used to measure the absorption spectra of GNRs and GNBs.

### Cell culture and cytotoxicity experiments

The HepG2 cells used for the cell culture experiments which were procured from the Cell Laboratory of the Cell Resource Center of the Chinese Academy of Sciences. Before the experiment, a certain concentration of HepG2 cells was seeded in a 96-well plate and placed in an incubator (37 °C, 5%CO2) for 12 h. The experimental method for testing the activity of HepG2 cells was similar to that described in a previous study^42^. For the detailed measurement method, please refer to Supplementary Material.

### Cellular uptake of GNRs and GNBs

Inductively coupled plasma mass spectrometry (ICP-MS) (ICAP-qc, Thermo Fisher, Germany) was used to measure the uptake of GNRs and GNBs by a single HepG2 cell^43^ The number of gold nanoparticles in a single cell could be obtained by dividing the test results of ICP-MS by the cell density in the sample and then by the mass of a single gold nanoparticle.

TEM (JEOL-1400HR, JEOL, Japan) was used to observe the distribution of GNRs and GNBs in HepG2 cells. Please refer to Supplementary Material for the description of specific experimental methods.

### Numerical simulation

The finite element method software developed by COMSOL Multiphysics (www.comsol.com) was employed to simulate the distribution of electric field and thermal response of gold nanoparticles excited by Gaussian light. In COMSOL simulations, the water around the gold nanoparticles was set to a spherical shape with a radius of 1700 nm. The water was divided into three layers; the outermost layer was set as an infinite element layer with a thickness of 500 nm to simulate thermal conduction in an infinite space, and the second outer layer was a perfectly matched layer with a thickness of 500 nm to simulate light propagation in an infinite space. Non-uniform grids with the smallest grid of 1 nm were employed.

### Photothermal therapy Experiments

In the photothermal treatment experiment, the laser light emitted by a titanium: sapphire oscillator (Mira 900 S, Coherent, Santa Clara, California CA, USA) with a repetition frequency of 76 MHz was reflected to an inverted microscope (Axio Observer A1, Zeiss, Santa Clara) La, California CA, USA), and a 60× objective lens was used to focus and observe the target cells. The radius of the laser spot was about 1 μm. In the photothermal treatment experiment, the cells were incubated in a medium containing GNRs and GNBs for 24 h. Then, the medium containing GNRs and GNBs was removed, and a new medium without GNRs and GNBs was added to a Petri dish.

## Supplementary Information

Supplementary Information.

## Data Availability

The data that support the findings of this study are available from the corresponding author upon reasonable request.

## References

[1] Vigderman L, Zubarev ER (2013). High-yield synthesis of gold nanorods with longitudinal SPR peak greater than 1200 nm using hydroquinone as a reducing agent. Chem. Mater..

[2] Huang X, El-Sayed IH, Qian W, El-Sayed MA (2006). Cancer cell imaging and photothermal therapy in the near-infrared region by using gold nanorods. J. Am. Chem. Soc..

[3] Strabalak SE, Chen J, Au L, Lu X, Li X, Xia Y (2017). Gold nanocages for biomedical applications. Gold nanocages for biomedical applications. Adv. Mater..

[4] Wu Y, Ail RK, Chen K, Fang N, El-Sayed MA (2019). Gold nanoparticles in biological optical imaging. Gold nanoparticles in biological optical imaging. Nano Today.

[5] Gentilini C, Evangelista F, Rudolf P, Franchi P, Lucarini M, Pasquato L (2008). Water-soluble gold nanoparticles protected by fluorinated amphiphilic thiolates. J. Am. Chem. Soc..

[6] Wu Y, Ail RK, Dansby K, El-Sayed MA (2019). Improving the flow cytometry-based detection of the cellular uptake of gold nanoparticles. Anal. Chem..

[7] Deinavizadeh M, Kiasat A, Hooshmand N, Shafiei M, Sabaeian M, Mirzajani R, Zahraei SM, Labouta HI, El-Sayed MA (2021). Smart NIR-light and pH responsive doxorubicin-loaded GNRs@SBA-15-SH nanocomposite for chemo-photothermal therapy of cancer. Nanophotonics.

[8] Zeng Y, Zhang D, Wu M, Liu Y, Zhang X, Li L, Li Z, Han X, Wei X, Liu X (2014). Lipid-AuNPs@PDA nanohybrid for MRI/CT imaging and photothermal therapy of hepatocellular carcinoma. ACS Appl. Mater. Interfaces.

[9] Wu Y, Ali RK, Dong B, Han T, Chen K, Chen J, Tang Y, Fang N, Wang F, El-Sayed MA (2018). Gold nanorod photothermal therapy alters cell junctions and actin network in inhibiting cancer cell collective migration. ACS Nano.

[10] Yuan H, Khoury CG, Wilson CM, Grant GA, Bennett AJ, Vo-Dinh T (2012). In vivo particle tracking and photothermal ablation using plasmon-resonant gold nanostars. Nanomed. Nanotechnol. Biol. Med..

[11] Gupta N, Malviya R (2021). Understanding and advancement in gold nanoparticle targeted photothermal therapy of cancer. Biochim. Biophys. Acta Rev. Cancer.

[12] Zhang Z, Wang J, Nie X, Wen T, Ji Y, Wu X, Zhao Y, Chen C (2014). Near infrared laser-induced targeted cancer therapy using thermoresponsive polymer encapsulated gold nanorods. J. Am. Chem. Soc..

[13] Goncalves DPN, Rodriguez RD, Kurth T, Bray LJ, Binner M, Jungnickel C, Gür FN, Poster S, Schmidt TL, Zahn DRT, Theotokis AA, Schlierf M, Werner C (2017). Enhanced targeting of invasive glioblastoma cells by peptidefunctionalized gold nanorods in hydrogel-based 3D cultures. Acta Biomater..

[14] Yougbaré S, Mutalik C, Krisnawati DI, Kristanto H, Jazidie A, Nuh M, Cheng T-M, Kuo T-R (2020). Nanomaterials for the photothermal killing of bacteria. Nanomaterials.

[15] Li X, Takashima M, Yuba E, Harada A, Kono K (2014). PEGylated PAMAM dendrimer-doxorubicin conjugate-hybridized gold nanorod for combined photothermal-chemotherapy. Biomaterials.

[16] Parchur AK, Sharma G, Jagtap JM, Gogineni VR, LaViolette PS, Flister MJ, White SB, Joshi A (2018). Vascular interventional radiology-guided photothermal therapy of colorectal cancer liver metastasis with theranostic gold nanorods. ACS Nano.

[17] Mutalik C, Okoro G, Krisnawati DI, Jazidie A, Rahmawati EQ, Rahayu D, Hsu W-T, Kuo T-R (2022). Copper sulfide with morphology-dependent photodynamic and photothermal antibacterial activities. J. Colloid Interface Sci..

[18] Huang X, Jain PK, El-Sayed IH, El-Sayed MA (2002). Plasmonic photothermal therapy (PPTT) using gold nanoparticles. Lasers Med. Sci..

[19] Karam JA, Ahrar K, Matin SF (2011). Ablation of kidney tumors. Surg. Oncol. Clin. N. Am..

[20] Yougbaré S, Mutalik C, Okoro G, Lin I-H, Krisnawati DI, Jazidie A, Nuh M, Chang C-C, KuoInt T-R (2021). Emerging trends in nanomaterials for antibacterial applications. J. Nanomed..

[21] Mutalik C, Okoro G, Chou H-L, Lin I-H, Yougbaré S, Chang C-C, Kuo T-R (2022). Phase-dependent 1T/2H-MoS2 nanosheets for effective photothermal killing of bacteria. ACS Sustain. Chem. Eng..

[22] Sugiyama K, Sakai T, Fujishima I, Ryu H, Uemura K, Yokoyama T (1990). Stereotactic interstitial laser-hyperthermia using Nd-YAG laser. Stereotact. Funct. Neurosurg..

[23] Zhang P, Cai T, Zhou Q, She G, Liang W, Deng Y, Ning T, Shi W, Zhang L, Zhang C (2022). Ultrahigh modulation enhancement in all-optical Si-based THz modulators integrated with gold nanobipyramids. Nano. Lett..

[24] Chen S, Wang Y, Liu Q, Shi G, Liu Z, Lu K, Han L, Ling X, Zhang H, Cheng S, Ma W (2018). Broadband enhancement of PbS quantum dot solar cells by the synergistic effect of plasmonic gold nanobipyramids and nanospheres. Adv. Energy. Mater..

[25] Kou X, Ni W, Teung CK, Chan K, Lin HQ, Stucky GD, Wang J (2007). Growth of gold bipyramids with improved yield and their curvature-directed oxidation. Small.

[26] Feng J, Chen L, Xia Y, Xing J, Li Z, Qian Q, Wang Y, Wu A, Zeng L, Zhou Y (2017). Bioconjugation of gold nanobipyramids for SERS detection and targeted photothermal therapy in breast cancer. ACS Biomater. Sci. Eng..

[27] Zhang M, Zhang H, Feng J, Zhou Y, Wang B (2020). Intracellularly generated immunological gold nanoparticles for combinatorial photothermal therapy and immunotherapy against tumor. Chem. Eng. J..

[28] Wilhelm S, Tavares AJ, Dai Q, Ohta S, Audet J, Dvorak HF, Chan WCW (2016). Analysis of nanoparticle delivery to tumours. Nat. Rev. Mater..

[29] Curcio JA, Petty CC (1951). The near infrared absorption spectrum of liquid water. J. Opt. Soc. Am..

[30] Segelstein DJ (1987). The Complex Refractive Index of Water.

[31] Mousavi SA, Malerod L, Berg T, Kjeken R (2004). Clathrin-dependent endocytosis. Biochem. J..

[32] Majno G, Joris I (1995). An overview of cell death. Am. J. Pathol..

[33] Hatef A, Deeschenes SF, Boulais E, Lesage F, Meunier M (2015). Photothermal response of hollow gold nanoshell to laser irradiation: Continuous wave, short and ultrashort pulse. Int. J. Heat Mass Transf..

[34] Govorov AO, Richardson HH (2007). Generating heat with metal nanoparticles. Nano Today.

[35] Johnson PB, Christy RW (1972). Optical constants of the noble metals. Phys. Rev. B..

[36] Chen X, Chen Y, Yan M, Qiu M (2012). Nanosecond photothermal effects in plasmonic nanostructures. ACS Nano.

[37] Gan R, Fan H, Wei Z, Liu H, Lan S, Dai Q (2019). Photothermal response of hollow gold nanorods under femtosecond laser irradiation. Nanomaterials.

[38] Hatef A, Darvish B, Burke A, Dagallier A, Meunier M (2016). Computational characterization of plasma effects in ultrafast laser irradiation of spherical gold nanostructures for photothermal therapy. J. Phys. D: Appl. Phys..

[39] Ekici O, Harrison RK, Durr NJ, Eversole DS, Lee M, Yakar AB (2008). Thermal analysis of gold nanorods heated with femtosecond laser pulses. J. Phys. D Appl. Phys..

[40] Ail MRK, Snyder B, El-Sayed MA (2012). Synthesis and optical properties of small au nanorods using a seedless growth technique. Langmuir..

[41] Li Q, Zhou X, Li S, Ruan Q, Wang XJ (2015). Production of monodisperse gold nanobipyramids with number percentages approaching 100% and evaluation of their plasmonic properties. Adv. Opt. Mater..

[42] Fan H, Le Q, Lan S, Liang J, Tie S, Xu J (2018). Modifying the mechanical properties of gold nanorods by copper doping and triggering their cytotoxicity with ultrasonic wave. Colloids Surface B.

[43] Zhou W, Liu X, Ji J (2012). More efficient NIR photothermal therapeutic effect from intracellular heating modality than extracellular heating modality: An in vitro study. Nanoparticle Res..

