# Supplementary material for: Highly localized, efficient, and rapid photothermal therapy using gold nanobipyramids for liver cancer cells triggered by femtosecond laser
Source: Sci Rep. 2023 Feb 27;13:3372. doi: 10.1038/s41598-023-30526-x (PMC9970969; doi:10.1038/s41598-023-30526-x)
Supplement: Supplementary file 1 — Supplementary Information. [file 41598_2023_30526_MOESM1_ESM.docx]

**Supporting Information**

**Highly Localized, Efficient, and Rapid Photothermal Therapy Using Gold Nanobipyramids for Liver Cancer Cells Triggered by Femtosecond Laser**

Xiao Liu^1†^, Wei Zhou^1†^, Tianjun Wang^2^, Sen Miao^1^, Sheng Lan^1^, Zhongchao Wei^1^, Zhao Meng^3^, Qiaofeng Dai^1*^, Haihua Fan^1*^

^1^ Guangdong Provincial Key Laboratory of Nanophotonic Functional Materials and Devices School of Information and Optoelectronic Science and Engineering, South China Normal University, Guangzhou 510006, China

^2^ School of Biological Science and Medical Engineering, Southeast University, Nanjing 210096, China

^3^ Guangdong Women and Children Hospital, Guangzhou 51000, China

^*^ Correspondence: [daiqf@scnu.edu.cn](mailto:daiqf@scnu.edu.cn); [fanhh@scnu.edu.cn](mailto:fanhh@scnu.edu.cn)

^†^ X.L. and W.Z. contributed equally to this work.

1. Synthesis Methods
   1. Synthesis of Polyethylene Glycol (PEG)-Coated Gold Nanorods (GNRs)

10 mL, 1 mM aqueous solution of tetrachloroauric acid trihydrate (HAuCl4·3H2O) was first added into 10 mL, 0.2 M cetyltrimethylammonium bromide (CTAB) solution at room temperature of 25~30℃. Then 500 μL, 4 mM of silver nitrate (AgNO3) solution was added into the mixed solution, followed by shaking gently. HCl (37%, 8 μL) was added to adjust the pH of the solution. After that, 140 μL, 78.8 mM ascorbic acid (AA) solution was added to the solution with gentle shaking until the solution was clear. Immediately afterward, 30 μL of freshly prepared 0.01 M sodium borohydride (NaBH4) was added into it, and allowed to react for 6 hours. After the reaction was completed, the supernatant containing CTAB was removed by high-speed centrifugation, and then deionized water was added for high-speed centrifugal cleaning, so that after repeated centrifugal cleaning 3 times, the precipitate was dispersed in deionized water. In order to obtain Polyethylene glycolated gold nanorods (PEG-GNRs), the above-mentioned GNRs after repeated centrifugal cleaning were redispersed in a PEG (1%) aqueous solution, and stirred overnight.

1.2 Synthesis of Polyethylene Glycol (PEG)-Coated Gold nanobipyramids (GNBs)

GNBs Growth : The Au NBP samples were prepared using the seed-mediated growth method. The seed solution was made by adding a freshly prepared, ice-cold 0.15 mL, 0.01 M NaBH4 solution to a mixed solution consisting of 0.125 mL, 0.01 M HAuCl4·3H2O, 0.25 mL, 0.01 M trisodium citrate and 9.625 mL deionized water under vigorous stirring. Then let the solution stand for reaction at least 2 hours at room temperature (28~30℃), and finally obtain orange-red gold seed solution. For the preparation of the growth solution, firstly, 2 mL, 0.01 M HAuCl4·3H2O, 0.4 mL, 0.01 M AgNO3 solution and 0.8 mL, 1 M HCl solution were added into 40 mL, 0.1 M CTAB solution in turn. Then 0.32 mL, 0.1 M of AA reagent was added and gently stirred until the solution was clarified to obtain a growth solution. The prepared gold seed solution (0.3-0.5 mL) was mixed with the growth solution, and then gently stirred for 10-60 seconds. Finally, the mixed solution was left standing overnight at room temperature (28~30℃). The relation of the stirring time of mixed solution of gold seed solution and growth solution on the longitudinal lengths of GNBs and the regulation of the volume of gold seed solution on the ultraviolet-visible absorption spectrum of GNBs are showed in Fig.S1.

GNBs Purification: As many gold nanospheres were produced in the synthesized GNBs solution, further purification of the above prepared GNBs solution was needed. The GNBs samples prepared above were centrifuged at 10000 rpm for 10 min. Then the supernatant was extracted, and the precipitate was dispersed with 30 mL, 0.08 M CTAC solution. After that 6 mL, 0.01 M AgNO3 solution and 3 mL, 0.1 M AA solution were added successively. The resulting solution was reacted in an oven at 65℃ for 4 hours, during which Ag overgrew on Au nanocrystals to produce bimetallic Au/Ag products, in which the volume of AgNO3 solution added to GNBs solution varied between 6 and 10 mL. After 4 h reaction, the bimetallic Au/Ag product was centrifuged at 8000 rpm for 10 min. After removing the supernatant, the precipitate was then dispersed in 30 mL, 0.05 M CTAB solution and stood at room temperature for about 4 h. During this period, Au/Ag heterooxides aggregated and precipitated to the bottom of the vessel, while spherical Au@Ag nanoparticles remained in the supernatant. The supernatant was discarded and the remaining Au/Ag isononane was re-dispersed in 20 mL of deionized water. The resulting solution was then mildly mixed with 0.4 mL, 30 wt % NH3·H2O and 0.3mL, 0.1M H2O2 and left standing for 4 h. During this process, the Ag segment was gradually etched away and AgCl precipitates were observed at the bottom of the container. Finally, the clarified supernatant was carefully removed and centrifuged at about 8000 rpm for 10 min. The precipitates were then dispersed in deionized water for further use. In order to obtain polyethylene glycolated GNBs (PEG-GNBs), the above centrifugally purified GNBs was re-dispersed in PEG (1%) aqueous solution and stirred overnight.


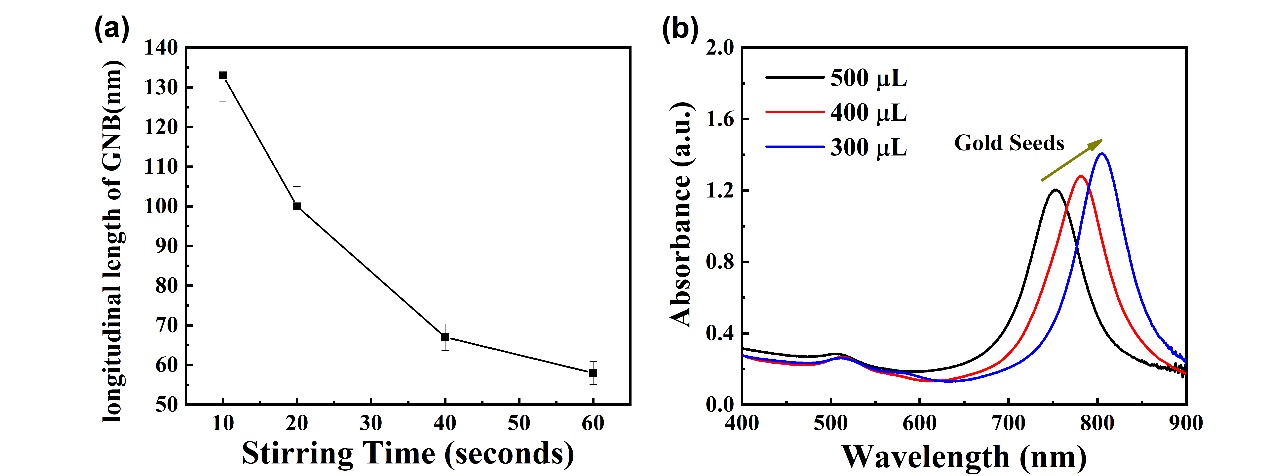


**Fig.S1** (a)the relation of the stirring time of mixed solution of gold seed solution and growth solution on the longitudinal lengths of GNBs (b)the regulation of the volume of gold seed solution on the ultraviolet-visible absorption spectrum of GNBs.

1. Expected distribution statistics of GNRs and GNBs

Fig.S2 shows the statistical graphs of the particle size distribution of GNRs and GNBs with different aspect ratios. Fig. S2(a) indicates that the average aspect ratio of GNRs is 3.50 with a standard deviation is 0.56, and Fig.S2(b) indicates that the average aspect ratio of GNRs is 3.71 with a standard deviation of 0.55; Fig.S2 (c) indicates that the average aspect ratio of GNBs is 2.92 with a standard deviation of 0.14; Fig.S2(d) indicates that the average aspect ratio of GNBs is 3.03 with a standard deviation of 0.17；


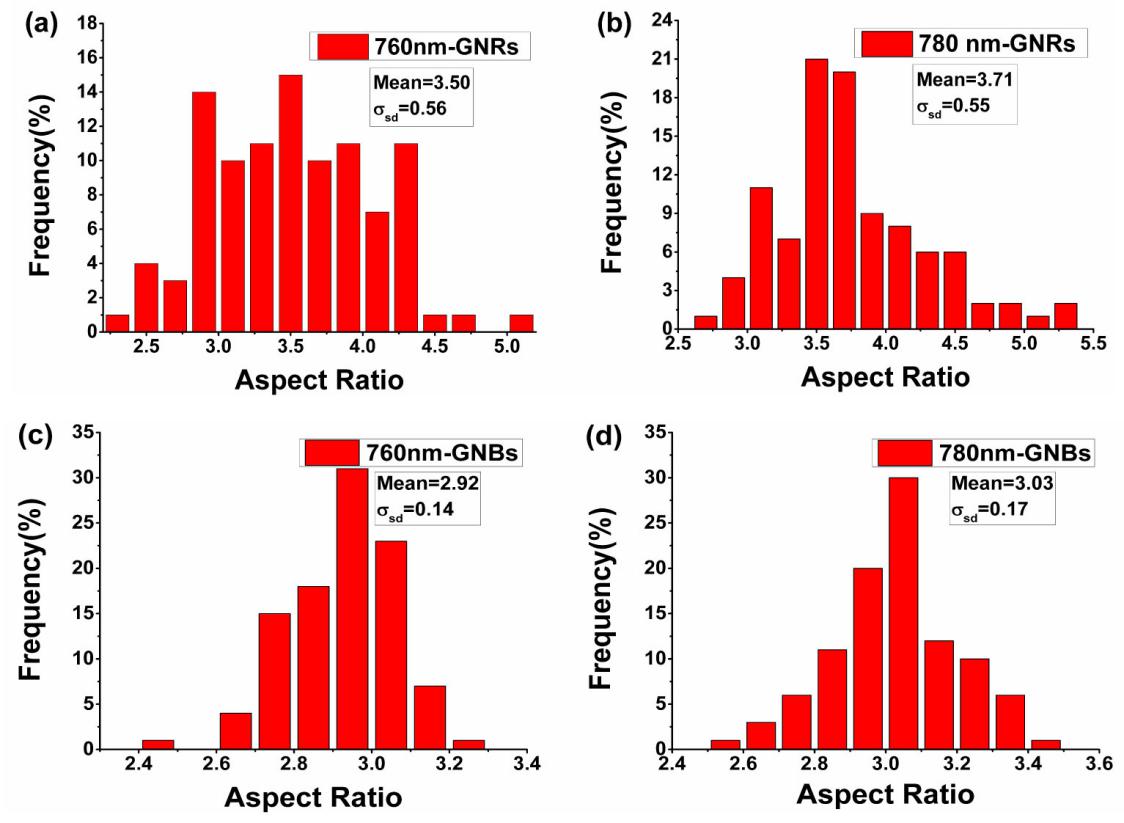


**Fig.S2** Particle size distribution of GNBs and GNRs of different sizes. (a) and (b) show the particle size distribution of GNRs with aspect ratios of 3.50 and 3.71, respectively. (c) and (d) show the particle size distribution of GNBs with aspect ratios of 2.92 and 3.03, respectively.Fluorescence emission spectra of GNRs and GNBs

3. Fluorescence emission spectra of GNRs and GNBs


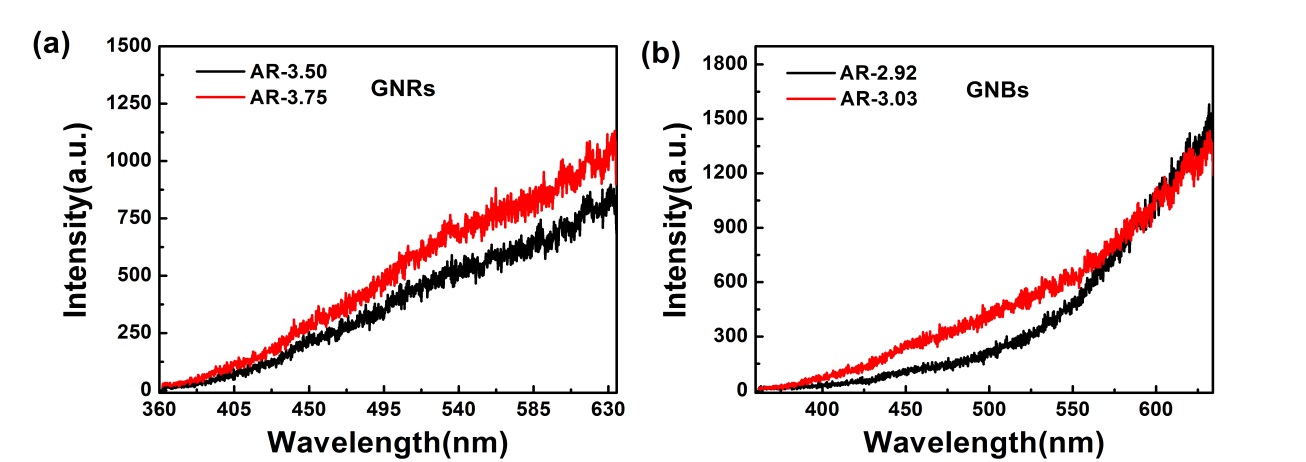


**Fig. S3** (a) fluorescence spectrum of GNRs with AR~3.50 excited by 760nm femtosecond laser and GNRs with AR~3.71 excited by 780nm femtosecond laser respectively (b): fluorescence spectrum of GNBs with AR~2.92 excited by 760nm femtosecond laser and GNBs with AR~3.03 excited by 780nm femtosecond laser respectively.

1. Cell culture and cytotoxicity experiments

In the cell viability test experiment, the MTT (thiazole blue) method was used for detection. Different concentrations of GNRs and GNBs were incubated with the experimental cells in the 96-well plate for 24 h, and then the absorbance of

96-well plate at 490 nm was measured using a microplate reader (BioRad). Calculate the survival rate of the cells through the calculation formula (2.1):

Cell viability=(ODtreated-ODblank)/(ODcontrol-ODblank)

In the above formula, OD refers to the absorbance of each well, the subscripts "treated" and "control" refer to HepG2 cells incubated with and without GNRs and GNBs respectively, and the subscript "blank" refers to the cell culture medium only.

1. Cellular Uptake of GNRs and GNBs

HepG2 cells were first co-incubated with GNRs and GNBs in an incubator (37°C, 5% CO2) for 24 h, then the cells were washed with phosphate buffer solution (PBS) for three times and centrifuged to form cell precipitates. After that, the precipitates were fixed with glutaraldehyde (2.5%), embedded in resin, and cut into ultra-thin sections. The samples were supported by a nickel mesh coated with an ultra-thin carbon film. Finally, the distribution images of GNRs and GNBs in cells were obtained under 200kV acceleration voltage.

1. Photothermal Therapy Experiments

The experimental device used in the photothermal therapy experiment is shown in Fig. S4.


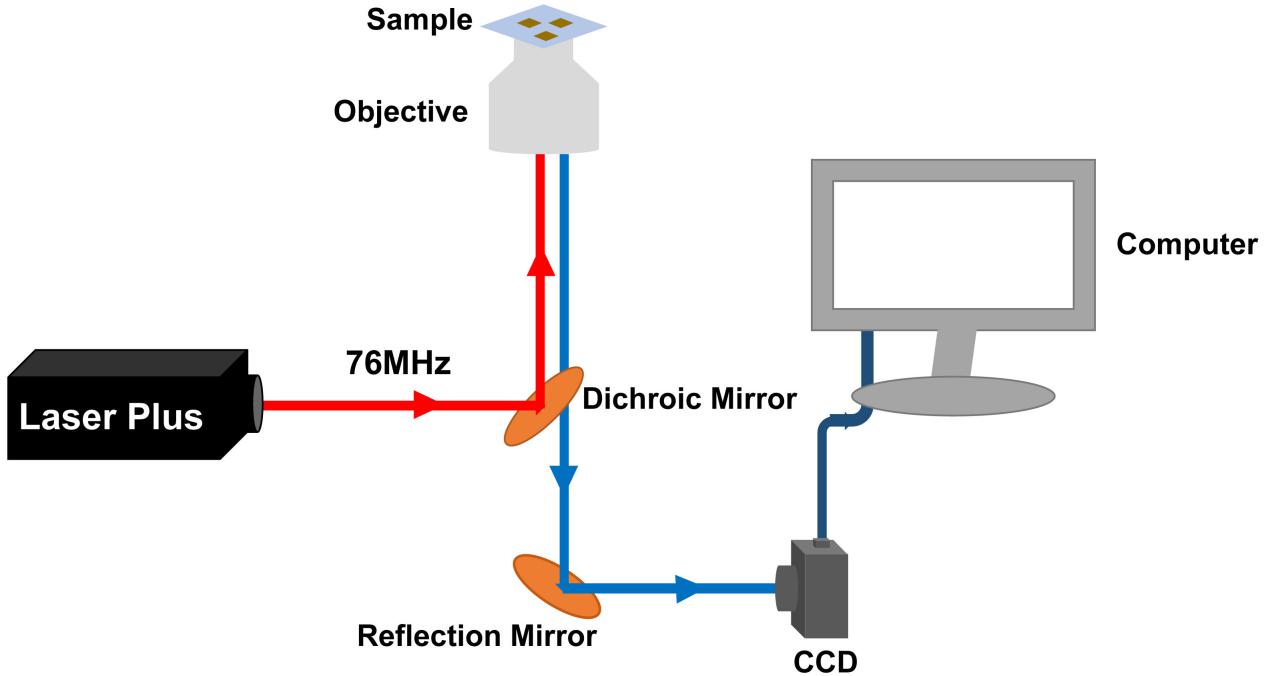


**Fig. S4** The experimental setup used in the photothermal therapy experiment.

1. Photothermal treatment of HepG2 cells by GNRs and GNBs under femtosecond pulsed laser


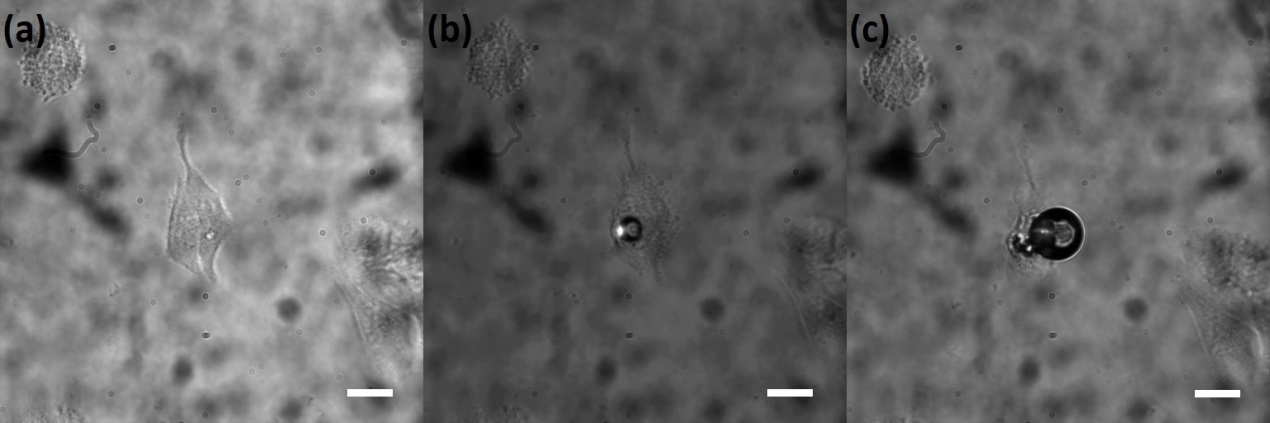


**Fig. S5** (a) cells morphology before laser treatm (b) during the laser treatment the laser focused onto the gold nanoparticle clusters, the bubble appeared.(c) as the laser treatment continued, the bubble volume increased. The length of thescale bar is 20μm.


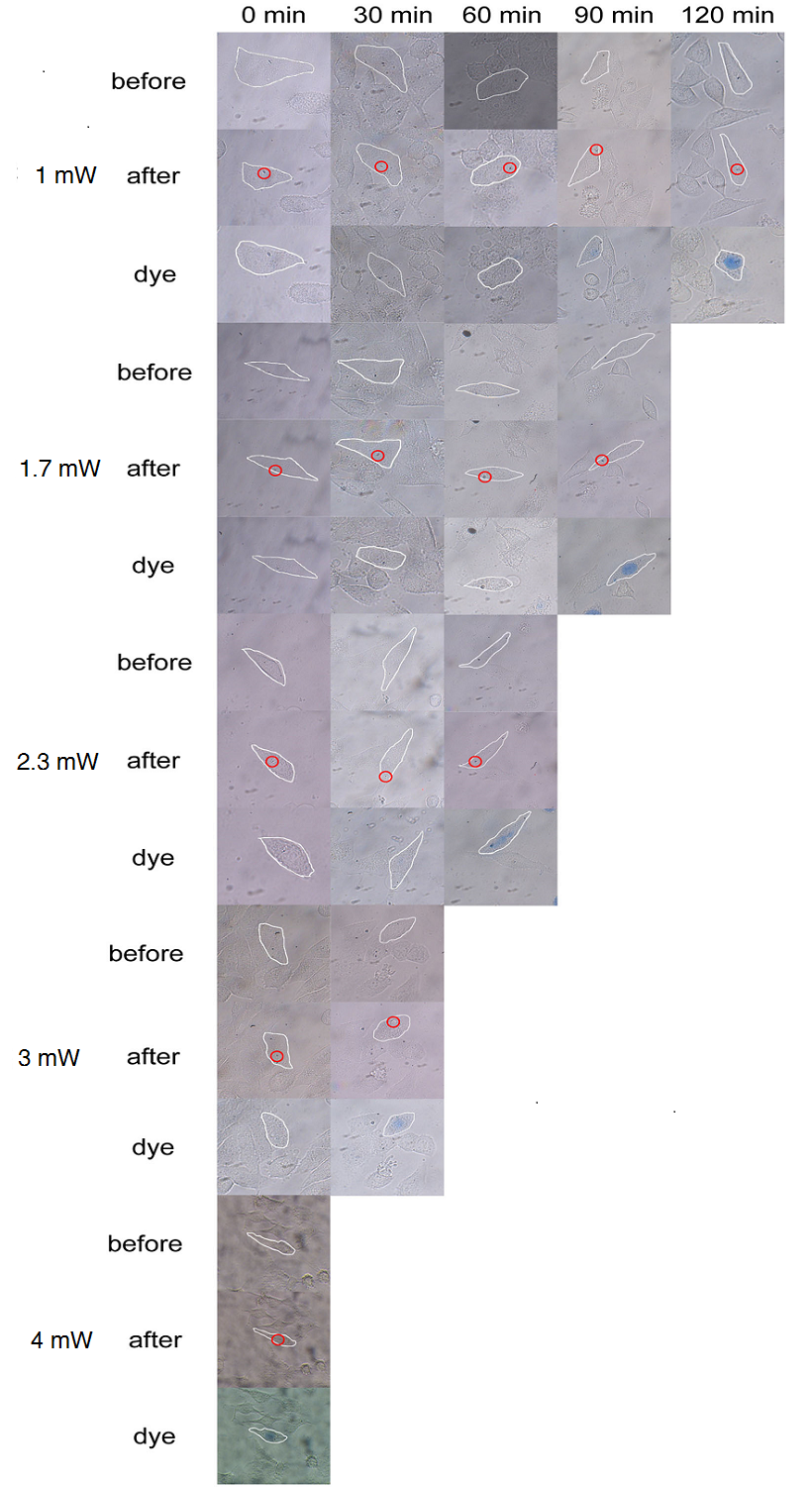


**Fig. S6** Photothermal treatment of GNRs with an aspect ratio of 3.50 under 760nm fs laser irradiation, the laser focuses on the nano-clusters in the cell. The red circle in the figure marks the position of the laser


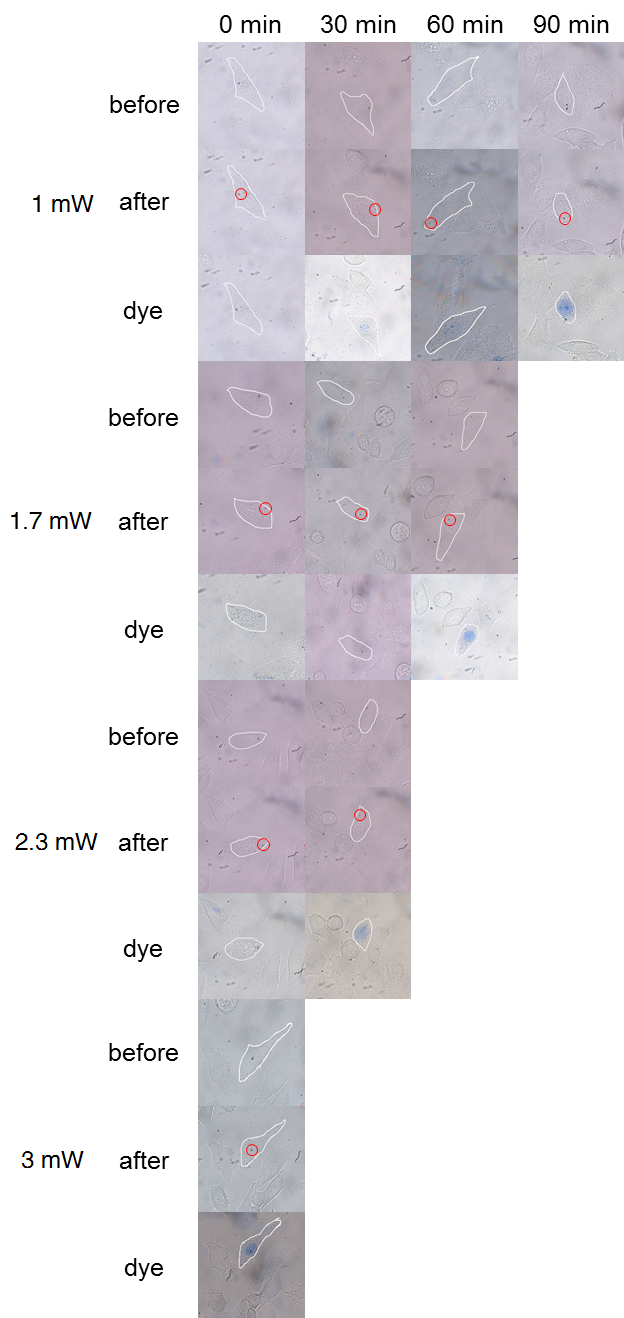


**Fig. S7** Photothermal treatment of GNBs with an aspect ratio of 3.03 under 780nm fs laser irradiation, the laser focuses on the nano clusters in the cell. The red circle in the figure marks the position of the laser


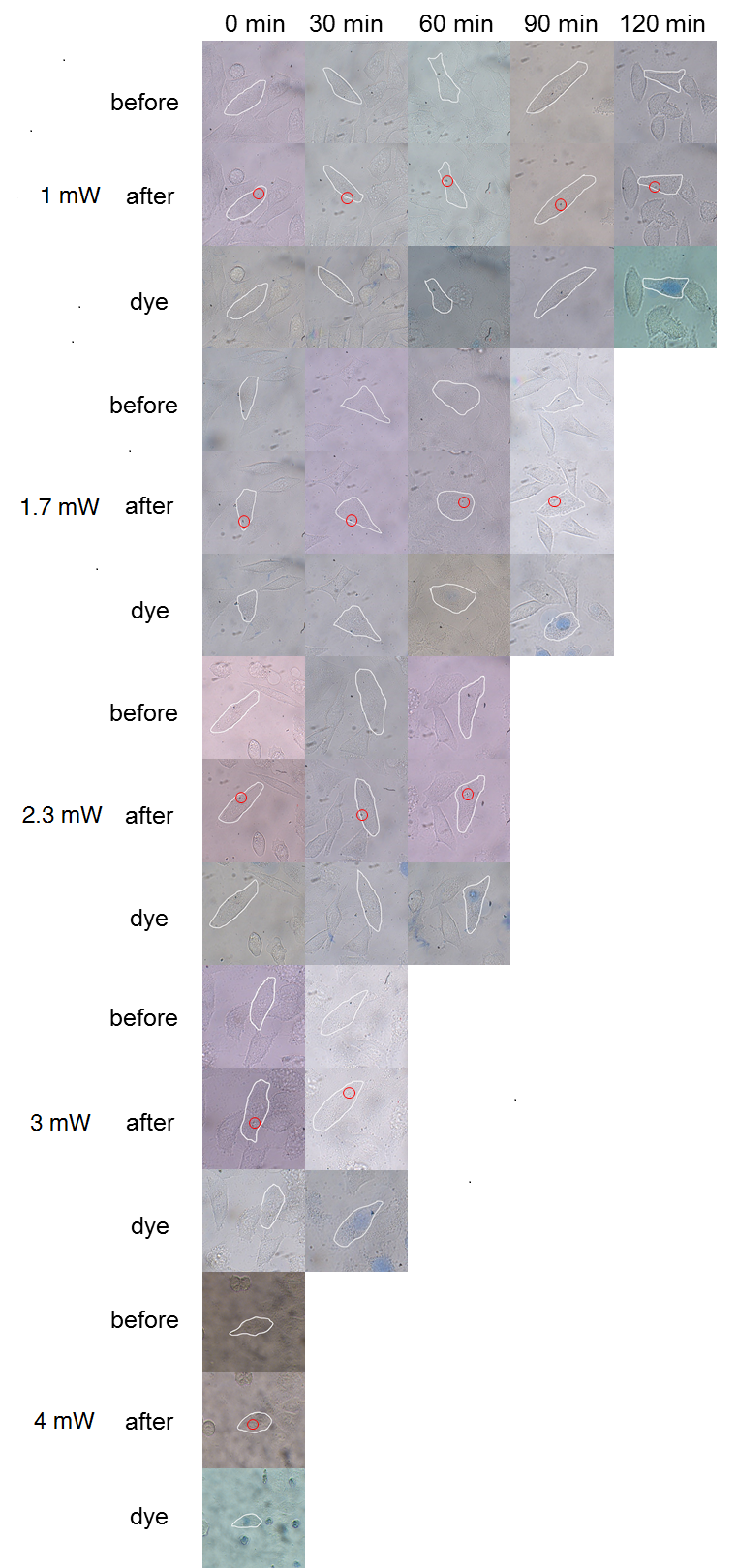


**Fig. S8** Photothermal treatment of GNRs with an aspect ratio of 3.71 under 780nm fs laser irradiation, the laser focuses on the nano clusters in the cell. The red circle in the figure marks the position of the laser


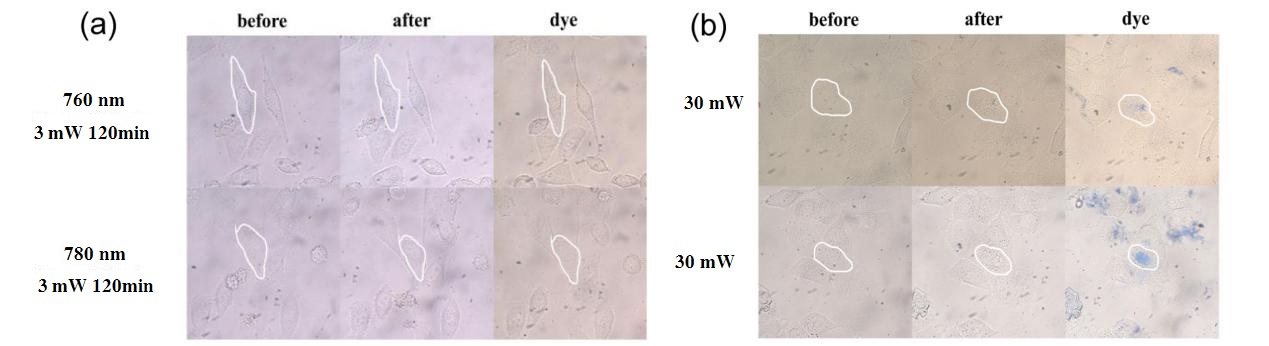


**Fig. S9** The image of HepG2 cells cultured without nanoparticles after being irradiated by fs laser. (a) the HepG2 cells irradiated by 3 mW fs laser for 20 s; (b) the HepG2 cells irradiated by 30 mW pulsed laser for 3 min.

1. GNBs and GNRs photothermal treatment of HepG2 cells under continuous laser


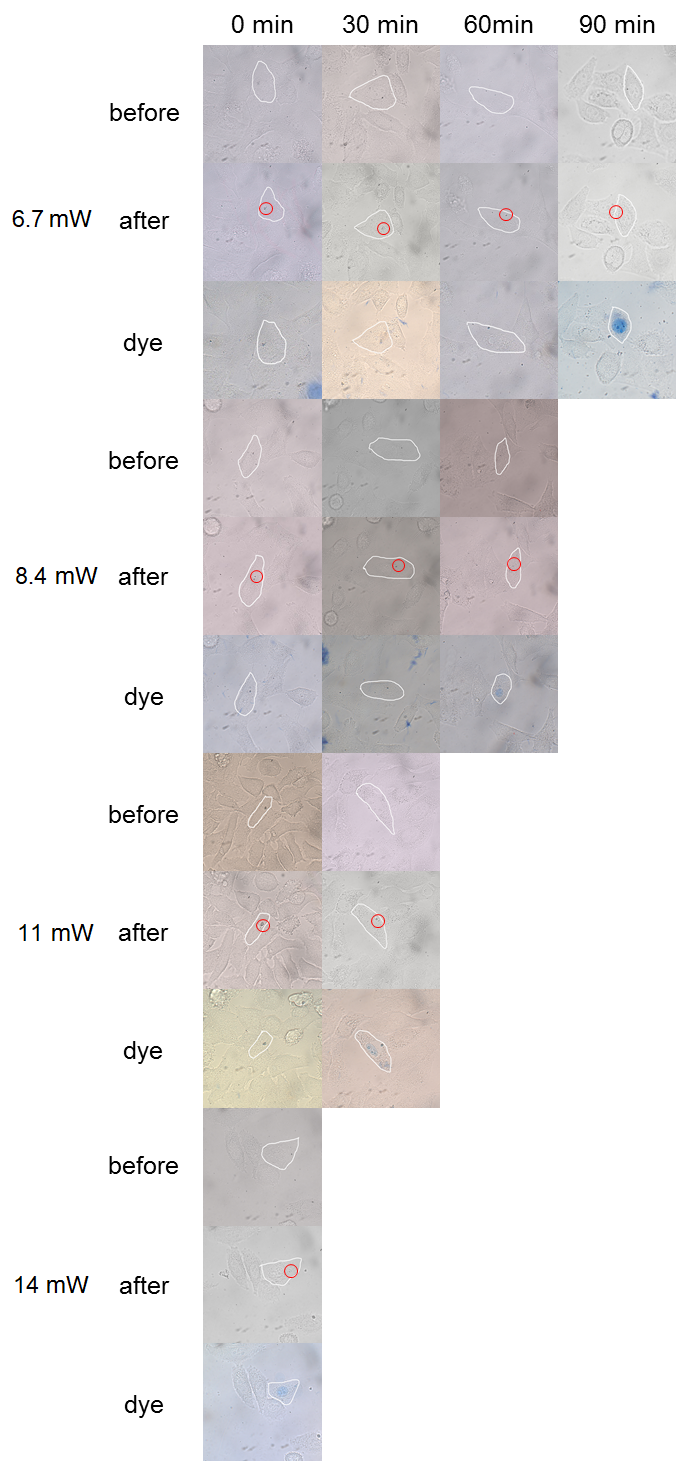


**Fig. S10** Photothermal treatment of GNRs with an aspect ratio of 3.50 under 760nm continuous laser irradiation, the laser focuses on the nano clusters in the cell. The red circle in the figure marks the position of the laser


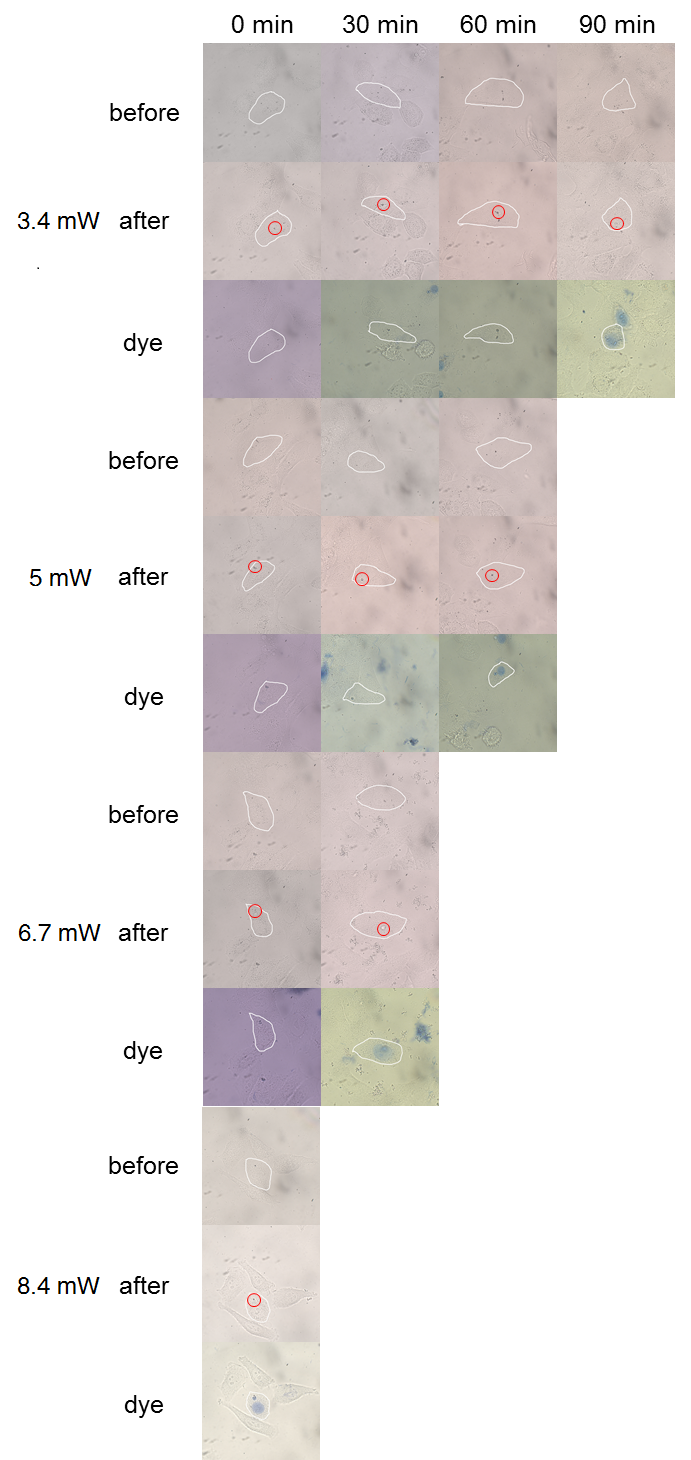


**Fig. S11** Photothermal treatment of GNBs with an aspect ratio of 3.03 under 780nm continuous laser irradiation, the laser focuses on the nano clusters in the cell. The red circle in the figure marks the position of the laser


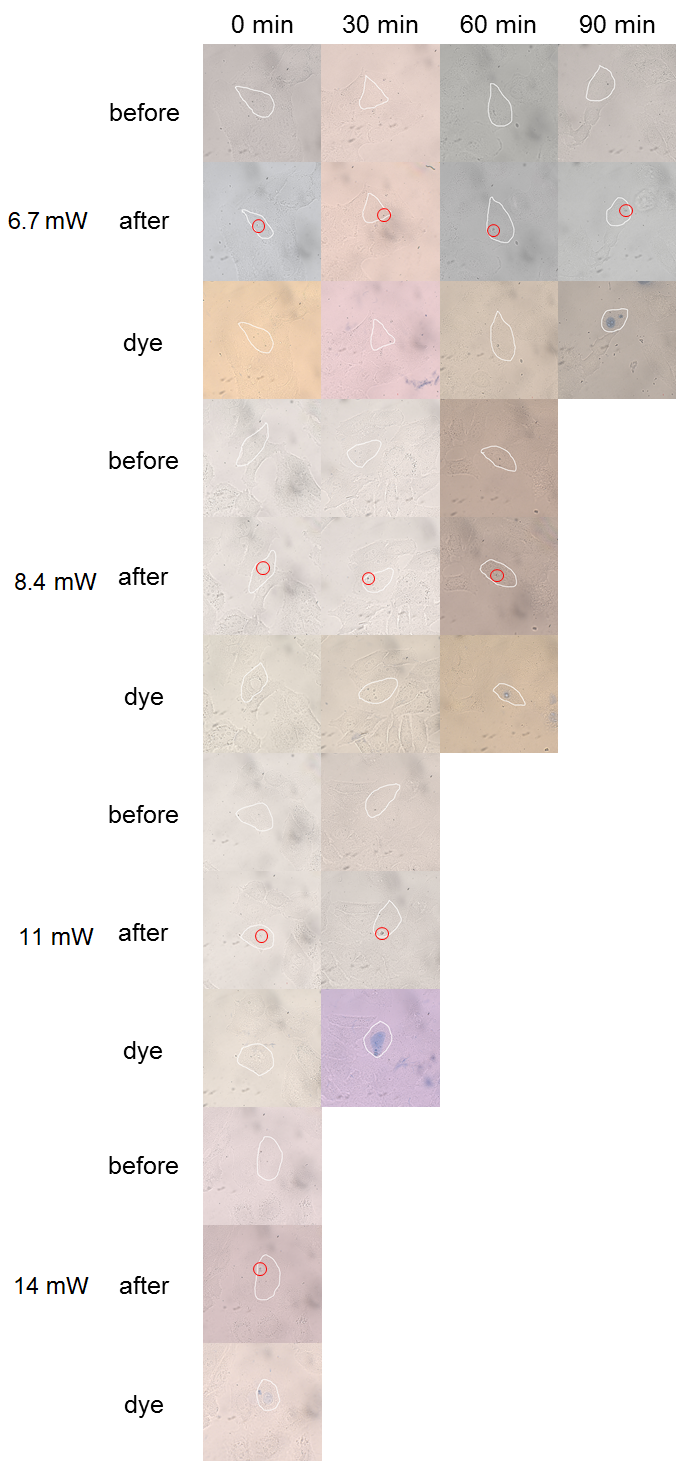


**Fig. S12** Photothermal treatment of GNRs with an aspect ratio of 3.71 under 780nm continuous laser irradiation, the laser focuses on the nano clusters in the cell. The red circle in the figure marks the position of the laser


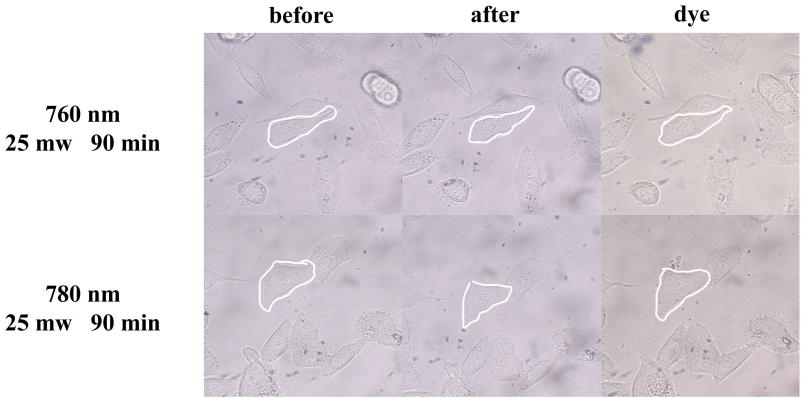


**Fig. S13** The image of HepG2 cells cultured without nanoparticles before and after being irradiated with 35 mw continuous laser for 5 minutes.

1. The form of cell death

Under the irradiation of fs pulse laser and continuous laser with different power, the death mode of cell death caused by GNBs and GNRs was studied, as shown in Fig. S14-S17. The fluorescence intensity values of control cells and experimental cells were listed in Table S1-Table S4 respectively.


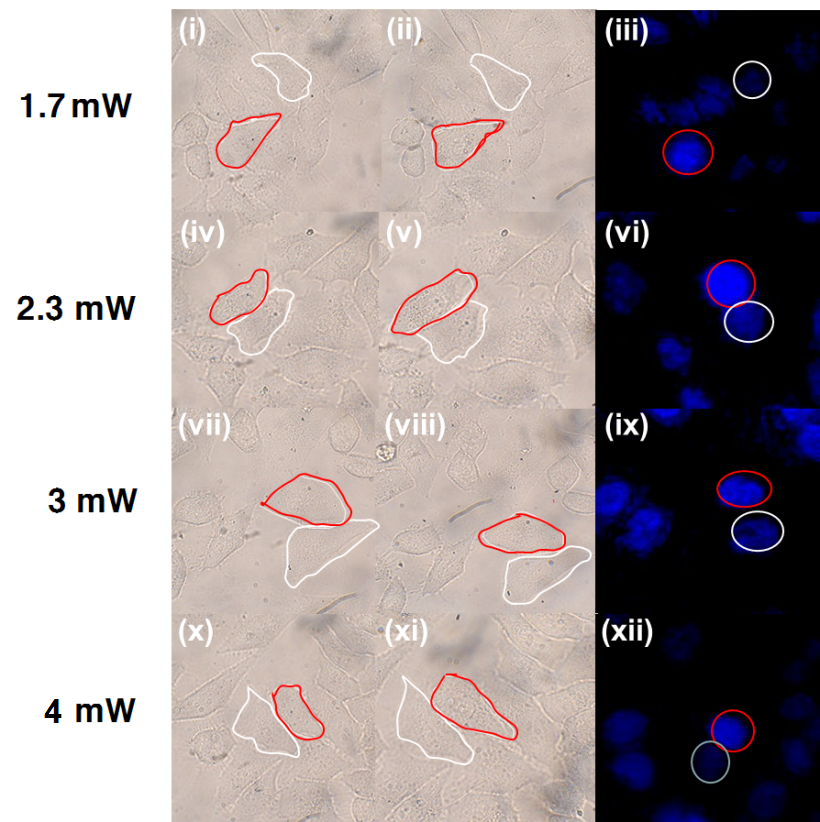


**Fig. S14** GNRs with an aspect ratio of 3.50 induce cell apoptosis under 760nm fs pulsed laser irradiation. The white circles represent control cells and the red circles represent experimental cells.


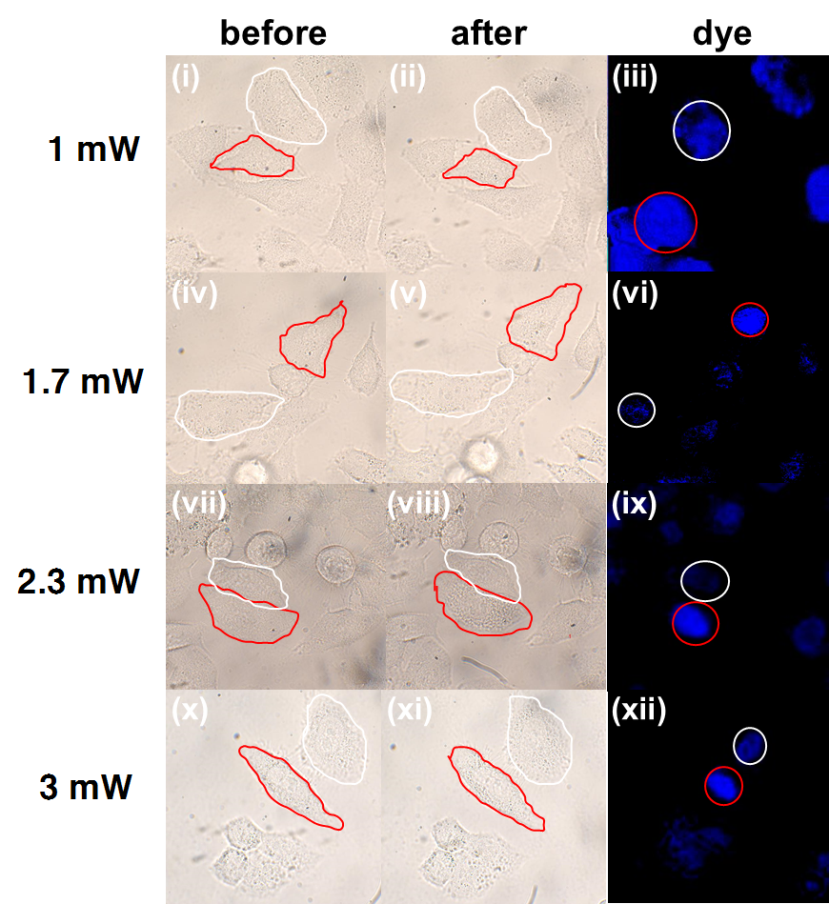


**Fig. S15** GNBs with an aspect ratio of 3.03 induce cell apoptosis under 780nm fs pulsed laser irradiation. The white circles represent control cells and the red circles represent experimental cells.


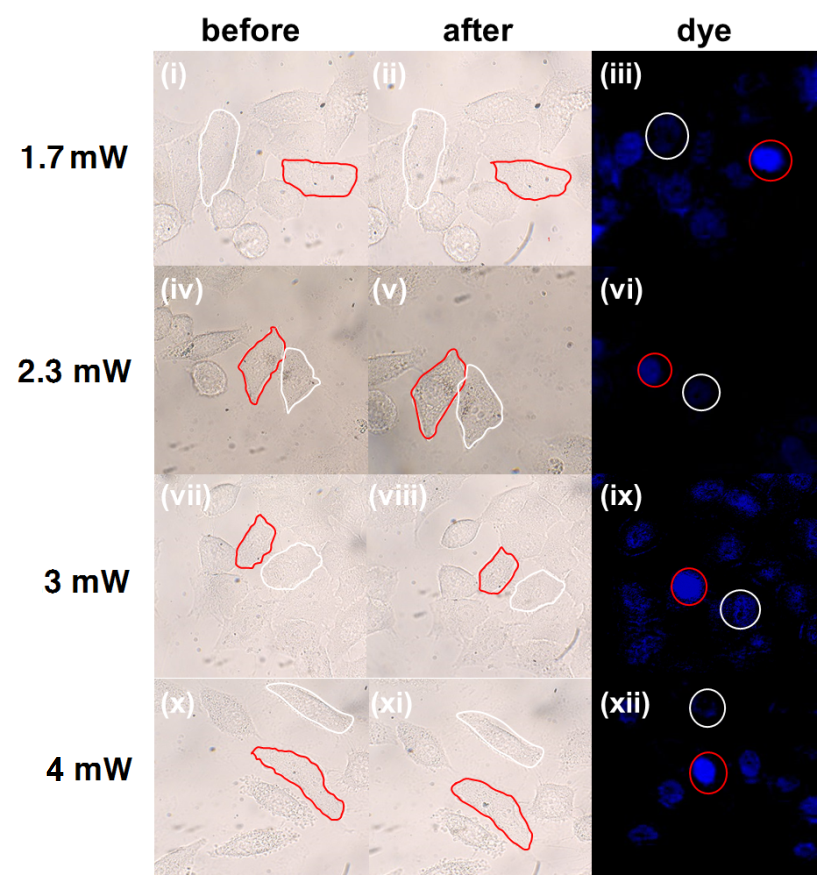


**Fig. S16** GNRs with an aspect ratio of 3.71 induce cell apoptosis under 780nm fs laser irradiation. The white circles represent control cells and the red circles represent experimental cells.


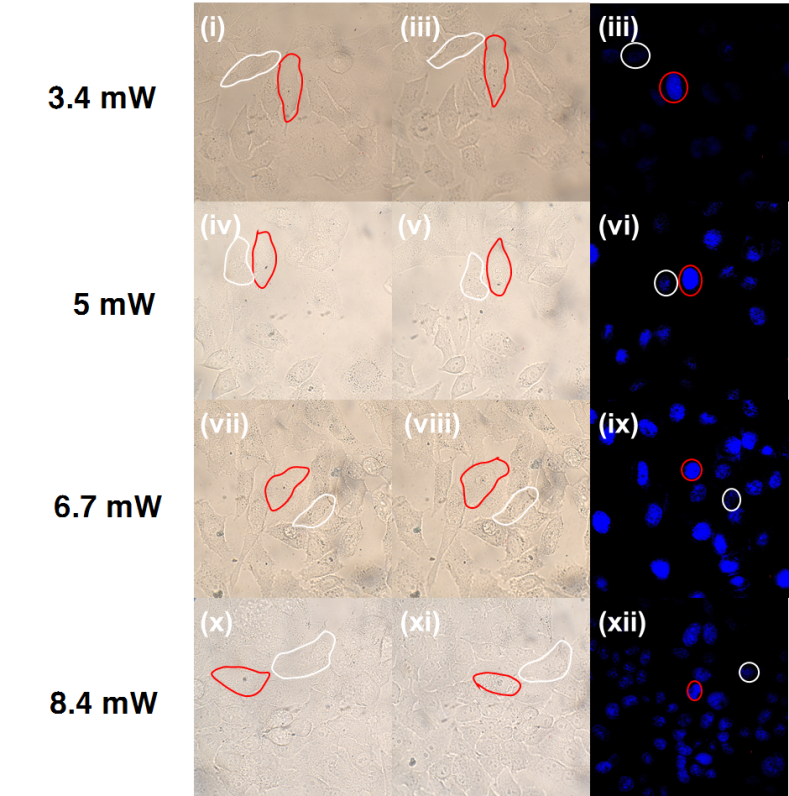


**Fig. S17** GNBs with an aspect ratio of 3.03 induce cell apoptosis under 780nm continuous laser irradiation. The white circles represent control cells and the red circles represent experimental cells.

**Table S1** Fluorescence intensity values of control cells and experimental cells (incubate with GNRs with an aspect ratio of 3.50) in Figure S13

| Sample | (iii)Control | (iii)GNRs | (vi)Control | (vi)GNRs | (ix)Control | (ix)GNRs | (xii)Control | (xii)GNRs |
| --- | --- | --- | --- | --- | --- | --- | --- | --- |
| Area(cell) | 1 | 1 | 1 | 1 | 1 | 1 | 1 | 1 |
| Intensity(mean) | 33.299 | 52.207 | 47.719 | 73.944 | 43.346 | 60.306 | 22.788 | 58.362 |

**Table S2** Fluorescence intensity values of control cells and experimental cells (incubate with GNBs with an aspect ratio of 3.03) in Figure S14

| Sample | (iii)Control | (iii)GNBs | (vi)Control | (vi)GNBs | (ix)Control | (ix)GNBs | (xii)Control | (xii)GNBs |
| --- | --- | --- | --- | --- | --- | --- | --- | --- |
| Area(cell) | 1 | 1 | 1 | 1 | 1 | 1 | 1 | 1 |
| Intensity(mean) | 28.200 | 49.754 | 14.027 | 29.920 | 37.445 | 54.647 | 34.960 | 69.140 |

**Table S3** Fluorescence intensity values of control cells and experimental cells (incubate with GNRs with an aspect ratio of 3.71) in Figure S15

| Sample | (iii)Control | (iii)GNRs | (vi)Control | (vi)GNRs | (ix)Control | (ix)GNRs | (xii)Control | (xii)GNRs |
| --- | --- | --- | --- | --- | --- | --- | --- | --- |
| Area(cell) | 1 | 1 | 1 | 1 | 1 | 1 | 1 | 1 |
| Intensity(mean) | 51.346 | 73.716 | 52.242 | 78.154 | 28.062 | 62.473 | 41.920 | 67.061 |

**Table S4** Fluorescence intensity values of control cells and experimental cells (incubate with GNBs with an aspect ratio of 3.03) in Figure S16

| Sample | (iii)Control | (iii)GNBs | (vi)Control | (vi)GNBs | (ix)Control | (ix)GNBs | (xii)Control | (xii)GNBs |
| --- | --- | --- | --- | --- | --- | --- | --- | --- |
| Area(cell) | 1 | 1 | 1 | 1 | 1 | 1 | 1 | 1 |
| Intensity(mean) | 35.063 | 51.346 | 64.449 | 83.494 | 46.284 | 64.211 | 41.920 | 82.470 |

1. Numerical simulation result of gold nanoparticle clusters


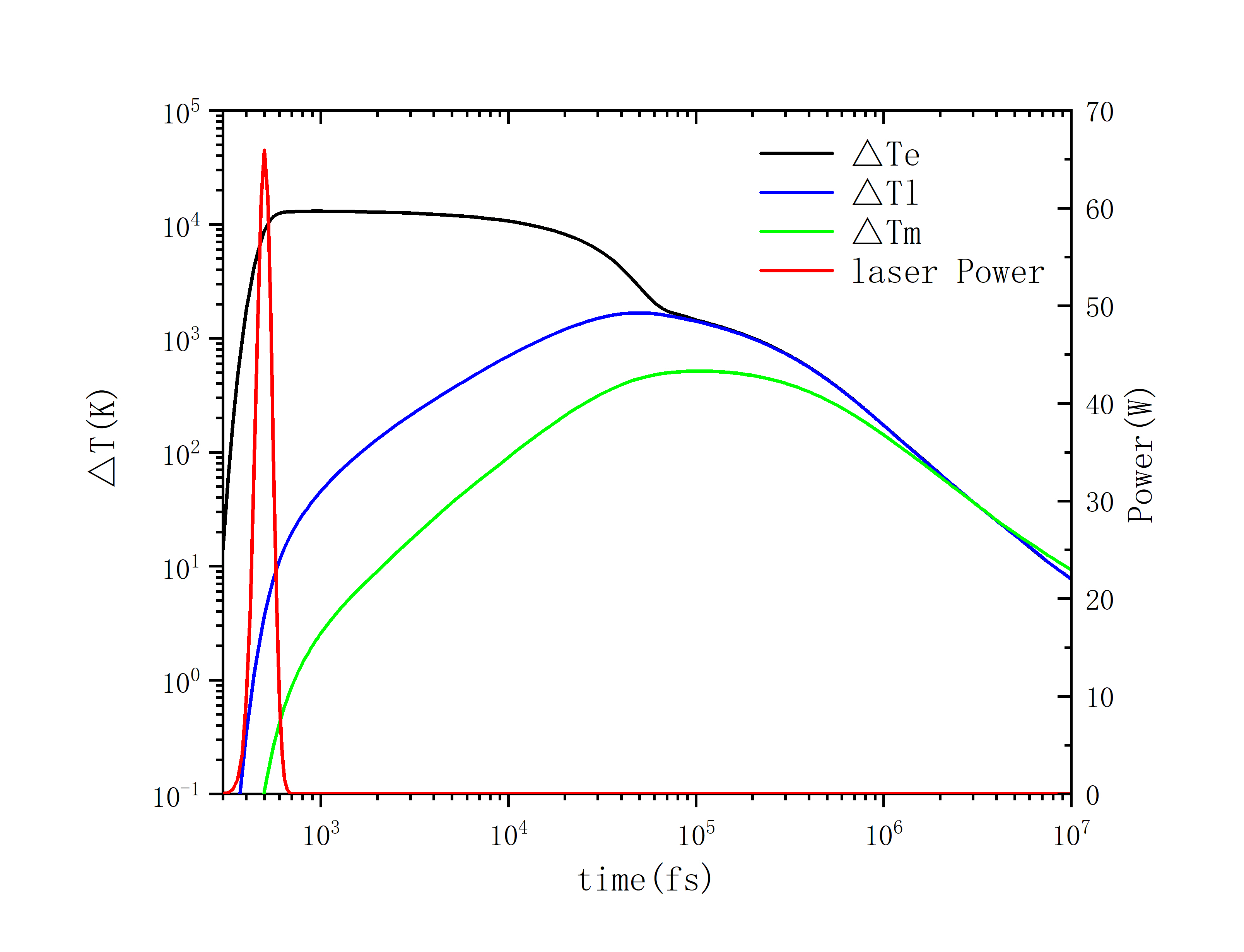


**Fig.S18.** Time dependent temperature evolution of electrons temperature Te, lattice temperature Tl and water temperature Tm at the GNB–media interface, for a laser pulse of 120 fs. The red curve shows the Gaussian intensity profile of the pulse. The max transient temperature increasement of water around GNB(LSPR 760 nm) clusters excited by 760nm fs laser in a pulse period (pulse width $t_{l}$=120fs, power =1mW, pulse frequency=76MHz).

1. Influence of nanoparticle density on photothermal temperature

The effect of density of nanoparticles on the temperature increase was studied and the results are shown in Fig.S19, taking the 760nm gold Nanobipyramids clusters in the text as an example, 10, 30, 70 gold nanoparticles are randomly extracted for simulation. Under the pulsed wave laser excitation, with the increase of particle density, the maximum temperature of water increases by 362 °C(Fig.S19a), 380 °C(Fig.S19b), 403 °C(Fig.S19c). It seems that the maximum temperature increase of water is proportional to the particle density, it is noted that in a pulse cycle, the maximum volume of the area with a temperature of more than 45 °C is 4.11×10^5^nm^3^, 5.16×10^5^nm^3^, 5.25×10^5^nm^3^. This shows that with the increase of particle density, the area with temperature more than 45 °C increases. Under continuous wave irradiation, with the increase of nanoparticle density, the maximum temperature increases by 147 °C(Fig.S19d), 166 °C(Fig.S19e), 212 °C(Fig.S19f), respectively, of which the temperature increase greater than 45 °C area volume is 4.1529×10^7^nm^3^, 1.9001×10^8^nm^3^, 5.7314×10^8^nm^3^, which indicates that the greater the particle density, the higher the temperature in the water and the larger the area with temperature exceeding 45°C. For pulsed laser, only one pulse period is considered in the simulation, and the heat generated is only near the surface of the nanoparticles, and does not spread farther, which can be seen from the pulsed photothermal area greater than 45 °C is 2~3 orders of magnitude lower than the continuous photothermal. After multiple pulse periods, as heat is generated and diffuse farther, more nanoparticles add heat in space result in higher temperature. But simulating multiple pulses is not feasible based on available computing resources. In general, the higher the density of nanoparticles, the more favorable it is to increase the local temperature required for photothermal therapy.


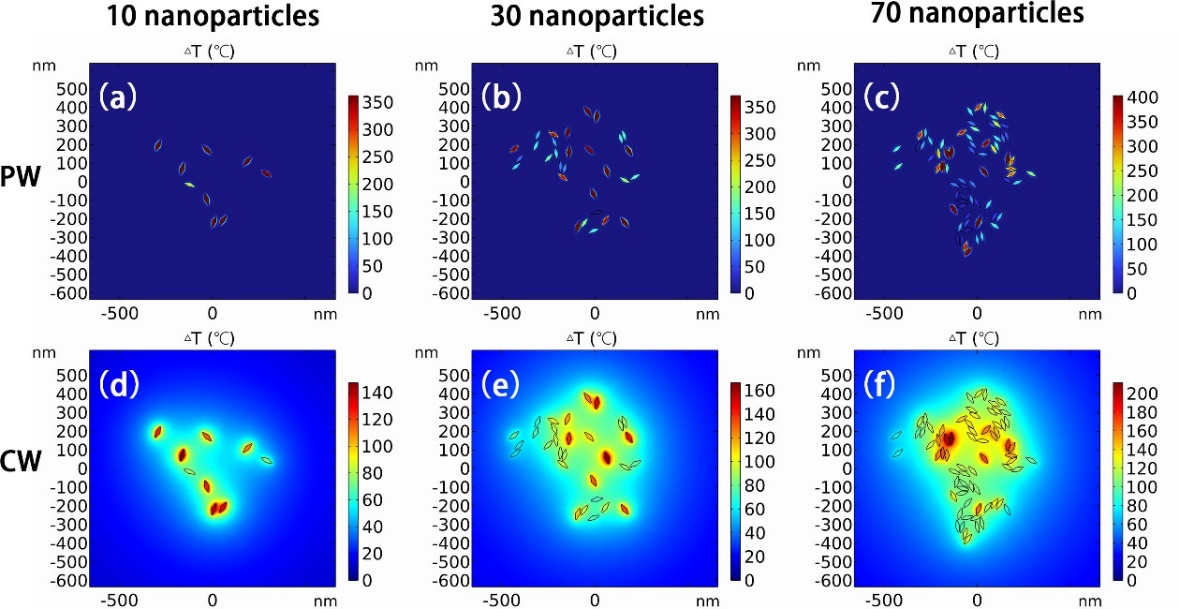


Fig.S19. Rising temperature distribution of GNB(LSPR 760nm) clusters under 760nm laser excitation (a) 10 nanoparticles, pulse fs laser excitation; (b) 30 nanoparticles, pulse fs laser excitation; (c) 70 nanoparticles, pulse fs laser excitation; (d) 10 nanoparticles, continuous laser excitation; (e) 30 nanoparticles, continuous laser excitation; (f) 70 nanoparticles, continuous laser excitation.
